# Intracellular localization of the mycobacterial stressosome complex

**DOI:** 10.1038/s41598-021-89069-8

**Published:** 2021-05-12

**Authors:** Malavika Ramesh, Ram Gopal Nitharwal, Phani Rama Krishna Behra, B. M. Fredrik Pettersson, Santanu Dasgupta, Leif A. Kirsebom

**Affiliations:** 1 Department of Cell and Molecular Biology, Biomedical Centre, Uppsala University, Box 596, 751 24 Uppsala, Sweden; 2 Present Address: Department of Biotechnology, Central University of Haryana, Mahendergarh, 123031 India

**Keywords:** Cell biology, Computational biology and bioinformatics, Microbiology, Molecular biology

## Abstract

Microorganisms survive stresses by alternating the expression of genes suitable for surviving the immediate and present danger and eventually adapt to new conditions. Many bacteria have evolved a multiprotein "molecular machinery" designated the "Stressosome" that integrates different stress signals and activates alternative sigma factors for appropriate downstream responses. We and others have identified orthologs of some of the *Bacillus subtilis* stressosome components, RsbR, RsbS, RsbT and RsbUVW in several mycobacteria and we have previously reported mutual interactions among the stressosome components RsbR, RsbS, RsbT and RsbUVW from *Mycobacterium marinum*. Here we provide evidence that "STAS" domains of both RsbR and RsbS are important for establishing the interaction and thus critical for stressosome assembly. Fluorescence microscopy further suggested co-localization of RsbR and RsbS in multiprotein complexes visible as co-localized fluorescent foci distributed at scattered locations in the *M. marinum* cytoplasm; the number, intensity and distribution of such foci changed in cells under stressed conditions. Finally, we provide bioinformatics data that 17 (of 244) mycobacteria, which lack the RsbRST genes, carry homologs of *Bacillus cereus* genes *rsbK* and *rsbM* indicating the existence of alternative σ^F^ activation pathways among mycobacteria.

## Introduction

Bacteria of the *Mycobacterium* genus inhabit various environmental reservoirs including soil, tap water, ground water and colonize insects, animals and humans^1,2^. Many of its members are pathogenic or opportunistic pathogens and can cause serious diseases such as tuberculosis (TB) and Hansen's disease (leprosy). In fact, roughly 30% of the more than 150 validated *Mycobacterium* spp. have been identified as potential animal and human pathogens^3–7^ (Pettersson et al., unpublished). Among the aquatic mycobacteria, *Mycobacterium marinum* (*Mmar*) causes mycobacteriosis, a TB-like disease, in fish^8,9^. It is a close relative of *Mycobacterium tuberculosis* (*Mtb*)^10^ and has emerged as a model organism for studying *Mtb* pathogenicity and physiology^11–13^.

Ability to adapt to new environments and/or novel metabolic requirements is a basic strategy for survival and bacteria have evolved various ways to survive different stresses. The molecular machinery involved in such processes are best characterized in the Gram-positive bacterium *Bacillus subtilis* in which specific sigma factors are activated in response to different stressful conditions. In *B. subtilis*, the Regulator of Sigma B (RsbRST) complex, also referred to as the "stressosome", is part of a signaling pathway that has an integrating role in stress response^14,15^. The *B. subtilis* 1.8 MDa pseudo-icosahedral RsbRST-complex comprises a core of multiple RsbR and RsbS subunits^16^ with the associated kinase RsbT acting as a switch; it dissociates from the core upon phosphorylation of RsbR and RsbS. The RsbR has a C-terminal "STAS" domain (Sulfate Transporter and Anti-Sigma factor antagonist domain), which interacts with another "STAS" domain in RsbS to form the core of the complex. In *B. subtilis*, RsbR is localized as punctuated foci scattered around the nucleoid in the cell and these foci do not dissociate upon stress induction^16,17^. Homologs of RsbRST-complex components have been identified in several bacteria and their gene organization on the chromosome is conserved^18^. However, many bacteria lack the RsbRST-complex but are still able to activate σ^B^ (or σ^B^ homologs)^19^ through alternative pathways.

Gene homologs to the RsbRST-complex and other proteins involved in activation of the alternative σ-factor σ^F^ (a homolog of both σ^B^ and the sporulation σ-factor σ^F^ in *B. subtilis*) exist in several mycobacteria^18–22^. We reported interactions among the proteins, included in the RsbRST-complex, that constitute the pathway leading to activation of σ^F^ as well as their expression under different growth conditions in *Mmar*^22^. Here we have performed deletion analyses and pull-down experiments to investigate which domains of RsbR and RsbS are important for the formation of the RsbRST-complex and the intracellular localization of the individual proteins and their putative complexes. Our data suggest that STAS domains are necessary for the interaction between RsbR and RsbS in *Mmar* and that RsbR and RsbS proteins need to be of full size in order to interact with RsbT in forming the RsbRST complex. The data from fluorescence microscopy suggest that RsbR and RsbS frequently co-localize as a single or multiple foci at the poles or are scattered in the cytoplasm of *Mmar* cells; the number and location of the foci changed as the cells moved from exponential to stationary phase*.* Bioinformatic analysis revealed the presence of the RsbRST module among the majority of the slow growing mycobacteria, SGM, while it is missing in the rapid growing mycobacteria, RGM, with a few exceptions. In this context, we present data suggesting the presence of genes supporting the existence of alternative σ^F^-activation pathways in mycobacteria.

## Results

### Bioinformatic analysis of σ^F^ activation in ***Mycobacterium*** spp

The *rsbR*, *rsbS* and *rsbT* genes encode the proteins forming the RsbRST-complex, the putative "stressosome" complex. These and other *rsb* genes are present in many but not all mycobacteria and 16S rRNA phylogeny revealed that species with a complete set of *rsb* genes cluster together on the phylogenetic tree^22^. We tested this conclusion further by plotting the core gene phylogenetic tree encompassing 244 mycobacterial genome sequences (unpublished data). This tree suggests that almost all mycobacteria, with the possible exceptions of *Mycobacterium franklinii*, *Mycobacterium stephanolepidis* and *Mycobacterium immunogenum* (all three are draft genomes), carry the genes encoding σ^F^ and anti-σ^F^ (*rsbW*; Fig. S1). Mycobacteria belonging to SGM harbor *rsbV*, *rsbR*, *rsbS*, *rsbT*, *rsbX* and *rsbUW* except members of the *Mtb* complex, *Mycobacterium leprae, Mycobacterium lepromatosis, Mycobacterium ulcerans*, and members of the *Mycobacterium terrae* and *Mycobacterium triviale* clades. Some of these genes are possibly also absent in some other SGM such as *Mycobacterium lepraemurium*. The majority of the RGM lack the *rsbR*, *rsbS*, *rsbT*, *rsbX* and *rsbUW* while *rsbV* is present in a number of RGM (Fig. S1). Moreover, *rsbR*, *rsbS*, *rsbT*, *rsbX* and *rsbUW* appear to have been acquired after the majority of the SGM group diverged from the RGM, *M. triviale* and *M. terrae* clades. In this context, members of the *Mycobacterium chelonae* clade, which represents the earliest mycobacterial lineage (Fig. S1), lack *rsbR*, *rsbS*, *rsbT*, *rsbX* and *rsbUW*, suggesting that these genes were acquired later during the evolution of the *Mycobacterium* genus. Several SGM and RGM also carry *rsbQ* and *rsbP* homologs, suggested to be involved in energy stress, as well as *rsbUVW* homologs (see e.g., Refs.^16,19,22^).

Alternatives to the "RsbRST pathway" for activation of σ^F^ were reported in, *e.g*., *Bacillus cereus* and *Streptomyces coelicolor*^19^. The RsbK, RsbM and RsbY proteins form the RsbMKY module in *B. cereus*. Together with RsbV, this module was suggested to affect RsbW and σ^F^ activities^23^. Homologs of RsbK and RsbM are present in *Mycobacterium gilvum*^19^ (Figs. S1-S3) and here we identified RsbK and RsbM homologs in 16 additional RGM strains, *e.g*., *Mycobacterium sphagni*, *Mycobacterium murale* and *Mycobacterium aurum* (Fig. S1). The gene synteny for *rsbK* and *rsbM* suggests that *rsbM* is positioned immediately downstream of *rsbK* (Fig. S2). Analysis of the genomes of *Mycobacterium vanbaalenii* (complete genome), *Mycobacterium austroafricanum* and *Mycobacterium vaccae* (the two latter draft genomes), which are phylogenetically close to *M. gilvum*, revealed that *rsbK* and *rsbM* are absent in *M. vaccae*. While in *M. vanbaalenii* and *M. austroafricanum rsbM* is present but they lack *rsbK* (Fig. S1) indicating variation (absence and presence of *rsbK* and *rsbM*) in closely related mycobacteria. Moreover, homologs to *rsbM* were also predicted to be present in several RGM and SGM (Fig. S1). For ten of these mycobacteria we could not predict the presence of "full-set" *rsbR*, *rsbS*, *rsbT*, *rsbX* and *rsbUW* while homologs to these genes were identified in *Mycobacterium moriokaense* and *Mycobacterium barrassiae* belonging to the *Mycobacterium gadium* clade (Fig. S1). It is therefore conceivable that the "RsbK-mediated" pathway of σ^F^-activation is operating in these species. However, note that we were unable to identify any homolog(s) for RsbY in mycobacteria. For those that lack the "RsbRST-mediated" and "RsbK-mediated" pathways (*e.g*., *M. vaccae*) σ^F^-activation might operate through a possible "RsbPQX-route"^19^.

Together these data suggest that there is some correlation between SGM and the presence of RsbRST as well as that one or more yet unknown alternative pathways exist for the regulation of SigF activity in mycobacteria.

### "STAS" domains and protein–protein interactions in the RsbRST-complex

**Figure 1:**
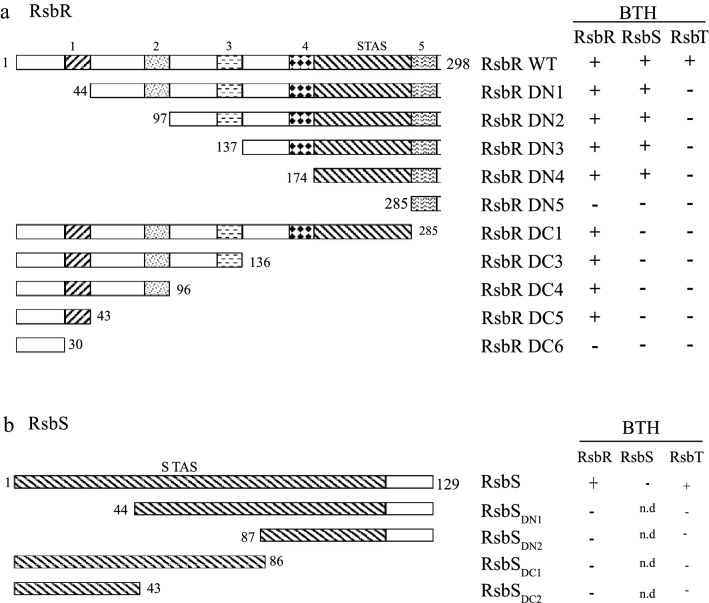
Schematic representation of domains of RsbR and RsbS proteins and design of several deletions along with the protein–protein interaction results of the bacterial two-hybrid (BTH/BACTH) assay. (**a**) The RsbR protein (298 amino acids long) contains a C terminal "STAS" domain (amino acids 174–285) and five (numbered 1 to 5) major coiled-coil (CC) regions distributed along the polypeptide as revealed using the "coils" tool available on Expasy. The amino acid numbers for start and end of ‘CC’ regions are as follows: 1. 30–43; 2. 83–96; 3. 123–136; 4. 159–173; 5. 275–289. Several RsbR deletions (names are given on the right-hand side of every scheme) are also shown along with their BTH (BACTH) interaction results with RsbR, RsbS and RsbT proteins. DN and DC refer to deletion at the N- and C-terminals, respectively. (**b**) The RsbS protein (129 amino acids long) mostly consists of a "STAS" domain (first 112 amino acids). The deletion scheme shows progressive 43 amino acids deletion either from N terminus (RsbS_DN1_ and RsbS_DN2_) or from the C terminus (RsbS_DC1_ and RsbS_DC2_). Right panel shows a summary of the BTH (BACTH) results. Note that full length RsbS is required for its interaction with RsbR and RsbT. In both panels A and B, plus and minus signs refers to interaction or no interaction based on Fig. S4. For details see main text.

*Mmar* RsbR and RsbS both have conserved "STAS" domains (Fig. 1)^18,24^. In the 298-unit polypeptide chain of RsbR, the "STAS" domain spans 111 amino acids at the C-terminal end (174 to 285; Fig. 1a) and closely resembles its homolog in *B. subtilis*. In contrast, the N-termini showed poor homology^18^. Several "coiled-coil" motifs (identified using the "coils" tool available on Expasy) were found distributed along the RsbR polypeptide making it "coiled-coil (CC)" rich, which might be significant for protein–protein interactions. We assigned five CC-regions numbered 1–5 to RsbR sequences, the start and end of which, were 30–43, 83–96, 123–136, 159–173 and 275–289, respectively (Fig. 1a). In this study, we focused on the *Mmar* strain CCUG20998^25^ hereafter referred to as *Mmar*^T^.

The RsbS "STAS" domain covers almost the entire protein (1–112 of the 129 amino acids long polypeptide chain) and does not show any CC region ("coiled-coil" motif; Fig. 1b). In order to identify the protein–protein interaction domains in RsbR and RsbS we generated a series of truncated N- and C-terminal variants (Fig. 1a,b). These constructs were tested for their ability to interact with themselves as well as with the other two components of the RsbRST complex using the bacterial two-hybrid system (BACTH), where blue colonies indicated that the two polypeptides did interact (Fig. S4a-d; summarized in Table 1). In agreement with Pettersson et al.^22^ the data suggested that intact RsbR and RsbS proteins interact with each other and with RsbT. The four N-terminal RsbR deletions (RsbR DN1-4), all of which have intact "STAS" domains, showed positive interactions with RsbS. Deletion of the RsbR "STAS" domain (DN5; which also includes amino acids belonging to the weak CC-region, positions 123–136) abolished its interaction with RsbS (Fig. S4a; see Supplementary information). These results suggested that the RsbR "STAS"-domain is important for forming the RsbR-RsbS complex, in agreement with what is known from the *B. subtilis* system^16^. The data further showed that none of the C-terminal truncated RsbR variants interacted with RsbS (including DC1, which retained almost an intact STAS domain; Fig. S4b) indicating that the RsbR CC-region 5 is also important for binding to RsbS. RsbR, but not RsbS, also interacts with itself^22^; however, large parts of RsbR from either N- or C-terminals including the "STAS" domain could be deleted without abolishing its self-interaction or oligomerization (Fig. 1a; Fig. S4a).

**Table 1:**
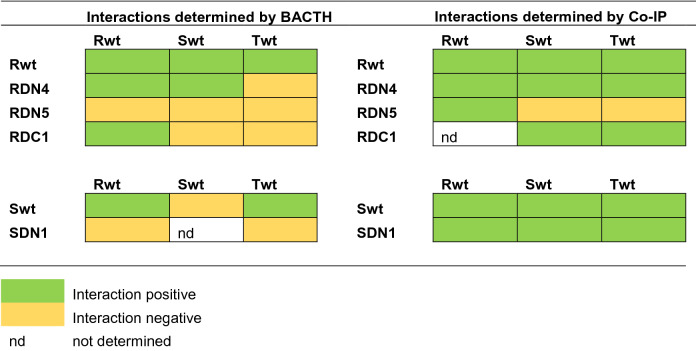
Summary of results from BACTH and Co-Immuno-precipitation (Co-IP) methods.

As shown in Fig. 1b, RsbS did not interact with itself; analysis of the truncated RsbS variants indicated that no regions of the protein could be deleted without affecting its interaction with RsbR (Fig. 1b; Fig. S4). These data indicated that the entire *Mmar*^T^ RsbS STAS-domain is required for binding to RsbR (but see below). Note that both full size RsbR and RsbS proteins were required for interaction with RsbT in forming RsbRT- and RsbST-complexes (Fig. 1a,b; Fig. S4c, d).

To validate the BACTH results, a co-immunoprecipitation (Co-IP) approach was performed (see Supplementary methods and constructions, Fig. S4e and f). In contrast to the BACTH results, all interactions except RsbS and RsbT with RsbRDN5 were detected to be positive (summarized in Table 1). However, we cannot exclude a positive interaction as the RsbRDN5-His is too small to be detected on the membrane: the anti-FLAG antibody failed to detect RsbRDN5-FLAG construct (Fig. S4g, top left blot) albeit it pulled down the RsbR-His interacting partner (Fig. S4g, bottom left blot). The RsbS-FLAG construct resulted in a weak but positive signal when probed with the anti-FLAG antibody (Fig. S4g, top left blot), while it pulled down RsbR-His (Fig. S4g, bottom left) with a stronger signal compared to the RsbR-His pulled down with the full-length RsbS-FLAG construct (Fig. S4g, top and bottom left blots). Compared to full-length RsbR detected with the RsbR-His fusions the appearance of smaller size bands might indicate that RsbR is exposed to cleavage at specific sites, perhaps due to protease cleavage (Fig. S4g, bottom panels). The simultaneous overexpression of RsbR, RsbS, or RsbT did not appear to influence the level of these smaller fragments. Moreover, the position of the fusion tags in the Co-IP assay (unlike for the BACTH assay) took into account the orientation of the RsbR, RsbS, and RsbT protein in the stressosome complex and this may explain the discrepancy between the results. Also, especially for weak interactions, the pull-down assay is better at detecting a positive signal with less background and BACTH can give false positives^26^.

The Co-IP experiment confirms the interactions between RsbR and RsbS, RsbR and RsbT, as well as between RsbS and RsbT indicated based on BACTH data. We also detected a self-interaction between RsbS proteins expected to be critical for RsbR dimerization and stressosome assembly^27^. The detection of additional interactions using Co-IP, compared to the BACTH assay, is possibly due to that the interacting proteins were co-expressed from a common promoter and using identical translation initiation regions (see Supplementary methods) to ensure as far as possible that both proteins were expressed at similar levels. While in the BACTH assay, the two interacting proteins were expressed from different plasmids with different copy numbers, which most likely resulted in different levels of the two proteins. This may therefore have masked some of the interactions, presumably the weaker ones. The BACTH and Co-IP data are summarized in Table 1.

We conclude that RsbR, RsbS, and RsbT can form the necessary interactions to form the stressosome. Moreover, based on the Co-IP data it appears that the RsbR STAS domain is essential for the formation of the RsbR-RsbS and RsbR-RsbT complexes while its absence does not prevent oligomerization of RsbR. The Co-IP data also indicated that deleting part of the RsbS STAS domain did neither prevent interaction with RsbR or RsbT, nor oligomerization of RsbS.

### Intracellular localization of RsbR and RsbS in *Mmar*^T^ cells

Results from BACTH-assay indicated that RsbR, RsbS and RsbT interact in STAS-dependent formation of putative stressosome complex(es). We attempted to have a visual demonstration of such associations in situ in *Mmar*^T^ by examining the intracellular locations of RsbR and RsbS and their co-localization, if any. These studies were performed in live cells with fluorescence-tagged proteins as well as with native proteins in fixed cells by immunolocalizations.

*(i) Fluorescence-tagged RsbR and RsbS in live cells*: We transformed *Mmar*^T^ with plasmids bearing in-frame fusion of the fluorescent proteins mCherry and GFP with RsbR and RsbS, respectively (see “Methods” and Supplementary information). The expression of the fused proteins and their presence in cells were confirmed by Western blot analysis of the cell lysates using anti-mCherry and anti-GFP antibodies. Signals at sizes corresponding to those expected for the fusion proteins were detected (Fig. S5: (a) RsbR-mCherry; (b) RsbS-GFP). However, the Western blots also showed bands corresponding to sizes of free mCherry and GFP proteins suggesting unexplained expression of the fluorescent proteins alone from the fusion constructs. It should be noted that the level of free GFP protein (2–6%) was much lower than that of free mCherry proteins (70–80%; compare panels A and B in Fig. S5). This was reflected in the relative frequencies of intracellular distributions of RsbR-mCherry and RsbS-GFP foci; number of cells bearing sharp foci were ≈10 × lower in the former (see below).

**Figure 2:**
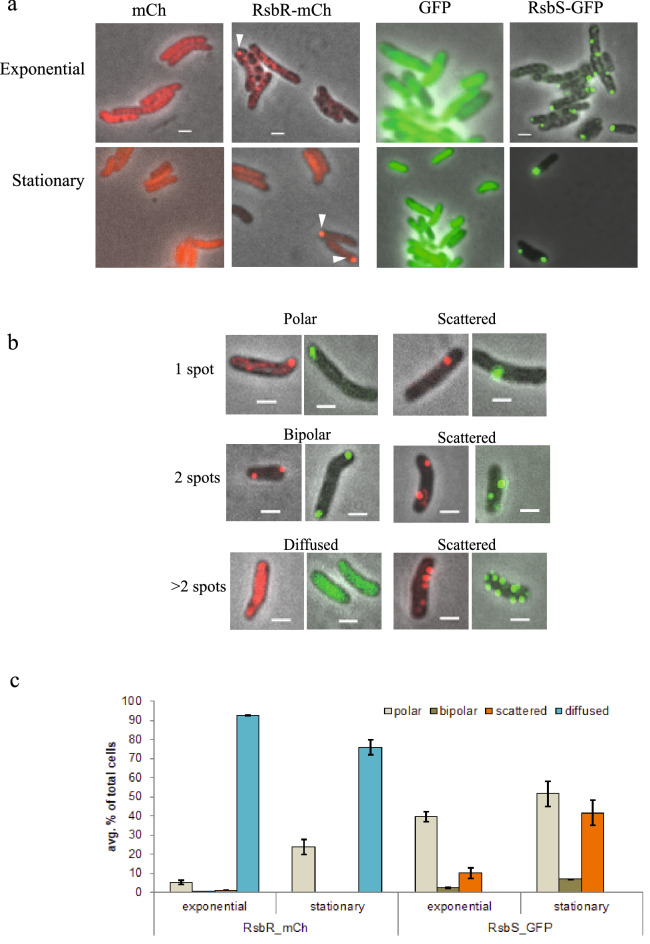
Localization of the "stressosome" subunits RsbR and RsbS in *Mmar*^*T*^ detected by mCherry and GFP fusions. (**a**) Represents *Mmar*^T^ strains carrying empty pBS401-mCherry (red) and pST2K-GFP (green) plasmids (first column to the left), the pBS401-RsbR-mCherry (red) and pST2K-RsbS-GFP (green) at exponential (2 days) and stationary phase (10 days) in second and third columns to the right. The foci of RsbR and RsbS localization is shown by white arrows, scale bar = 1 μm. (**b**) Represents the various classification of localization for statistics based on the position and number of spots (localizations) in a cell, scale bar = 1 μm. (**c**) Shows the average percentage distribution (with errors) of the different types of localizations of RsbR and RsbS in exponential and stationary growth phases with respect to total number of cells considered. As shown, most of the cells showed dispersed fluorescence with RsbR-mCherry in both exponential and stationary phase (blue bars). A total of two biological replicates were considered and minimum of 300 cells were counted for each time point/condition. The y-axis represents the average percentage of different "cell types" of total number of counted cells.

The locations of RsbR-mCherry and RsbS-GFP in live cells from exponentially growing and stationary phase cultures of *Mmar*^T^ carrying appropriate plasmids (see Supplementary information and Table S2) were visualized under a fluorescence microscope (Fig. 2a). The different columns show mCherry and GFP fluorescence in free state and in fused state (with RsbR and RsbS, respectively) at two growth phases, exponential phase (2 days, Fig. 2a top row) and stationary phase (10 days, Fig. 2a bottom row). Cells expressing mCherry or GFP proteins alone showed no localized foci but diffused fluorescence irrespective of growth phase, indicating that the dye proteins are expressed abundantly and spread uniformly throughout the cell interior. Both mCherry and GFP, fused to RsbR and RsbS, respectively, displayed sharp foci localized at poles as well as scattered in the cytoplasm (Fig. 2a). The foci for the RsbR-mCherry fusion were visible in less than 10% and 20% of exponentially growing and stationary phase cells, respectively (Table S5), of the cells with diffused fluorescence from free mCherry expression in the rest. In contrast, RsbS-GFP showed clear and sharp foci with almost none of the cells showing diffused fluorescence of free GFP (Fig. 2a; compare RsbR-mCherry and RsbS-GFP fluorescences). These were consistent with the difference in expression of the two fusion constructs as discussed above and shown in the Western blot (Fig. S5).

The number and intracellular locations of RsbR and RsbS foci in individual cells were classified in a few distinct types; Fig. 2b shows the representatives of these types, cells with 1, 2 and > 2 foci. In addition, there were cells with no foci but diffused fluorescence. The foci could be detected at one pole (polar), both poles of a cell (bipolar) or randomly distributed within the cytoplasm (non-polar or scattered). The foci were designated as polar when the signal intensity ratio between pole to mid-cell region was significantly higher compared to the same intensity ratio in cells expressing mCherry or GFP proteins alone (Fig. S6a; note the higher fluorescence intensity for RsbS-GFP focus compared to RsbR-mCherry).

Large number of cells (see Tables Supplementary information) were counted for each sample and the frequencies of intracellular locations of RsbR and RsbS foci are presented as histogram (Fig. 2c) and in tabulated form (Table S5). Almost all cells with dye alone show diffused fluorescence (90–100%) while the frequency of cells with distinct foci for RsbR-mCherry and RsbS-GFP ranged from 7 to 100%, depending on growth phase of the cells (Fig. 2c; Table S5). In exponentially growing cells, fluorescence from mCherry- and GFP-tagged RsbR and RsbS were located predominantly as a single sharp focus near one of the poles (75–80% of cells with foci) and the rest (20–25% of cells with foci) showed multiple (≥ 2) spots distributed within the cytoplasm. Despite the difference in the proportions of cells with mCherry and GFP foci (≈7% and ≈53%, respectively, of the total number of cells; Table S5), the relative frequencies of unipolar, bipolar and scattered foci distribution are quite similar for RsbR and RsbS (Table S5). For stationary phase cells grown for 10 days on plates cell poles appeared to be the preferred locations for RsbR and RsbS foci though in different proportions (Fig. 2c; Table S5). Noteworthy, the frequency of cells with RsbR and RsbS foci increased approximately two-fold in stationary phase relative to that in exponential phase (Table S5).

*(ii) Immunolocalization of native RsbR and RsbS proteins*: In order to verify whether the fused fluorescent proteins were affecting the intracellular locations of the RsbR and RsbS, their intracellular locations in native in situ forms were also examined by immunofluorescence microscopy using polyclonal antibodies raised against these two proteins. However, controls with pre-immune sera for RsbS showed a high frequency of false positive fluorescent foci (Fig. S6b); RsbS was therefore tagged with the FLAG octapeptide, (referred to as *Mmar*^pSTKiT-RsbS-FLAG^; see Supplementary Information for details) and anti-FLAG antibody was used to localize the RsbS protein in cells. Anti-RsbR antibody was used for detecting intracellular locations of RsbR.

**Figure 3:**
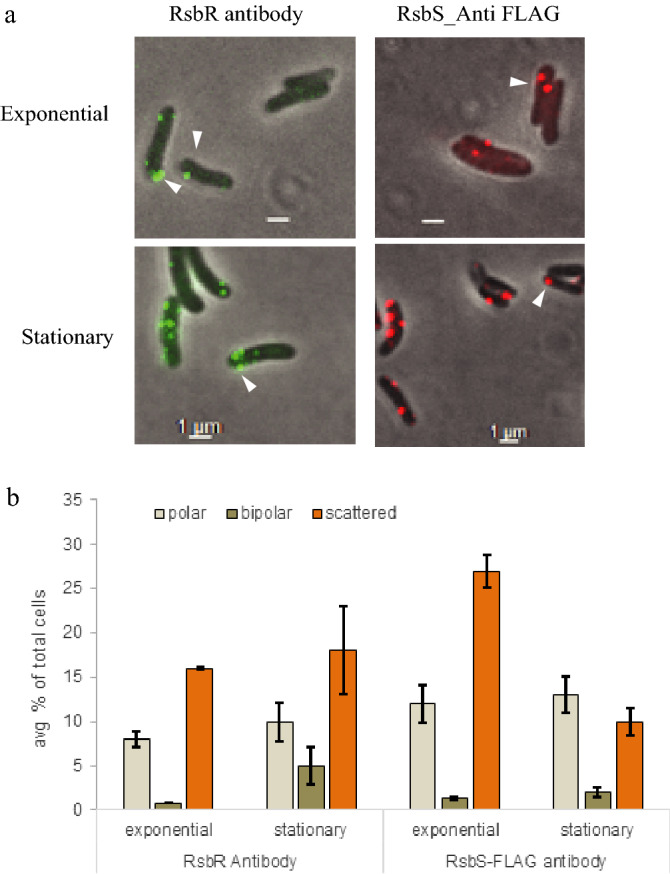
Localization of the "stressosome" subunits RsbR and RsbS in *Mmar*^T^ detected by Immunofluorescence. (**a**) Shows the RsbR (first column) and RsbS (second column) localization in *Mmar*^T^ and *Mmar*^pSTKiT-RsbS-FLAG^ cells at exponential (top panel) and stationary phase (bottom panel) by immunofluorescence technique using antibodies against RsbR and anti-FLAG for RsbS, respectively. White arrows mark localizations, scale bar = 1 μm. (**b**) Shows the average percentage distribution of the different types of localizations in the two phases (exponential and stationary phase) with standard deviations. A minimum of 900 cells were considered for each time point. The y-axis represents the average percentage of different "cell types" of total number of counted cells.

Figure 3a shows the intracellular locations of the native RsbR and RsbS-FLAG in cells from exponential (two day-old) and stationary (ten day-old) phase cultures. The proteins appear as sharp fluorescent foci both at cell poles as well as scattered in cytoplasm, as seen in live cells with the mCherry and GFP fusion constructs, without any diffused fluorescence in the background. Cells with RsbR and RsbS-FLAG fluorescent foci were categorized as before (Fig. 2b), counted and the frequencies of the different types were plotted as histograms (Fig. 3b) and presented in Table S6. The relative frequencies of RsbR and RsbS foci localized at poles and those scattered in cytoplasm were now different from those of the fluorescence-tagged proteins in live cells; *i.e*., more antibody-labeled foci were scattered in cytoplasm than localized at the poles (compare data Tables S5 and S6). Such quantitative differences could be attributed to the difference in assay methods since the concentrations of RsbR and RsbS in these two cases would be very different (see “Discussion” for an explanation of the difference between fluorescence tags and immunolocaliztion experiments). Also, it should be noted that the fluorescent foci, in cells labeled with either RsbR or RsbS fusion proteins or anti-RsbR or anti-FLAG antibody could represent locations of either RsbR and RsbS proteins or their oligomers, or some multiprotein complex such as RsbRS stressosome assembly.

**Figure 4:**
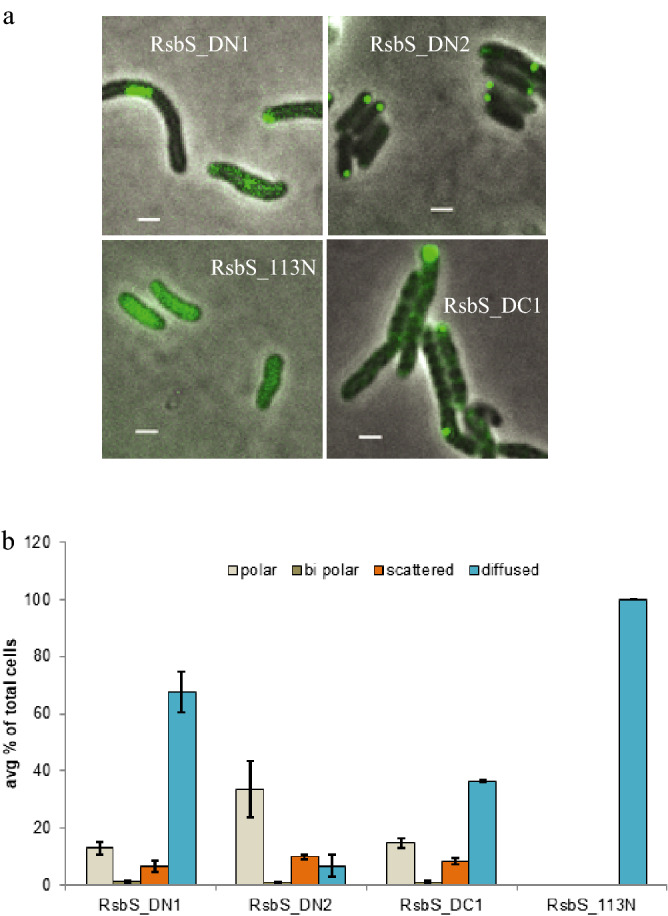
Intracellular localization of RsbS deletions. (**a**) Shows the RsbS localization in *Mmar*^pST2K-RsbS-GFP^ fusion cells with N and C terminal deletions of RsbS, scale bar = 1 μm. (**b**) Shows the average percentage distribution of the different types of localizations in the RsbS (N- and C-termini) deletion constructs in the exponential phase with standard deviations. A minimum of 500 cells were considered for each time point.

*(iii) Role of STAS and other regions on RsbR-RsbS interaction*: To examine how the absence of STAS domain of RsbS affected its location in the cell, we performed fluorescence microscopy of *Mmar*^T^ cells carrying GFP fusions of the deletion variants of RsbS: RsbS_DN1_, RsbS_DN2_, RsbS_DC1_ and a variant in which amino acid residues 1 to 112 were deleted (RsbS_113N_-GFP; see Fig. 1b). GFP fusion for RsbS_113N_ did not show any foci but diffused fluorescence distributed all over the cells while for RsbS_DN1_, RsbS_DN2_ and RsbS_DC1_ fluorescent foci were detected in cells at reasonably high frequencies (Fig. 4a,b). Detailed localization frequencies of foci from the deletion RsbS (RsbS_DN2_ and RsbS_DC1_-GFP) are given in Table S7 and shown as histogram (Fig. 4b). The results suggested that large parts of RsbS could be deleted without compromising its ability to form fluorescent foci or even their polar localization; RsbS_DN2_-GFP showed slightly lower polar localization frequency compared to the RsbS_wt_-GFP fusion, ≈66% vs. ≈76%, respectively (cf. exponential phase Figs. 2 and 4). However, deleting most of the "STAS" domain (RsbS_113N_) abolished foci formation almost completely (Fig. 4 and Table S7). Furthermore, since the deletion variants do not interact with RsbR (see Fig. 1b and Fig. S4), none of the foci represented RsbRS/RsbRST complexes but possible RsbS aggregates. Thus, the RsbR-RsbS interaction does not appear to be essential for either foci formation or their polar localization when overproduced from an extrachromosomal plasmid. This is consistent with the general observation that non-functional proteins show a tendency to end up near cell poles (see Discussion).

### Co-localization of RsbR and RsbS

The data presented so far show that the full size RsbR and RsbS proteins, fluorescence-tagged or in native form, could be located at cell poles as well as scattered in the cytoplasm; but (as discussed above), whether or which of these foci represented individual RsbR or RsbS or their oligomeric aggregates, or RsbR-RsbS complexes, could not be ascertained from labeling of single proteins. To distinguish between these alternatives, we attempted to label both RsbR and RsbS in the same cell and record overlapped or co-localized foci to represent RsbR-RsbS complex(es).

**Figure 5:**
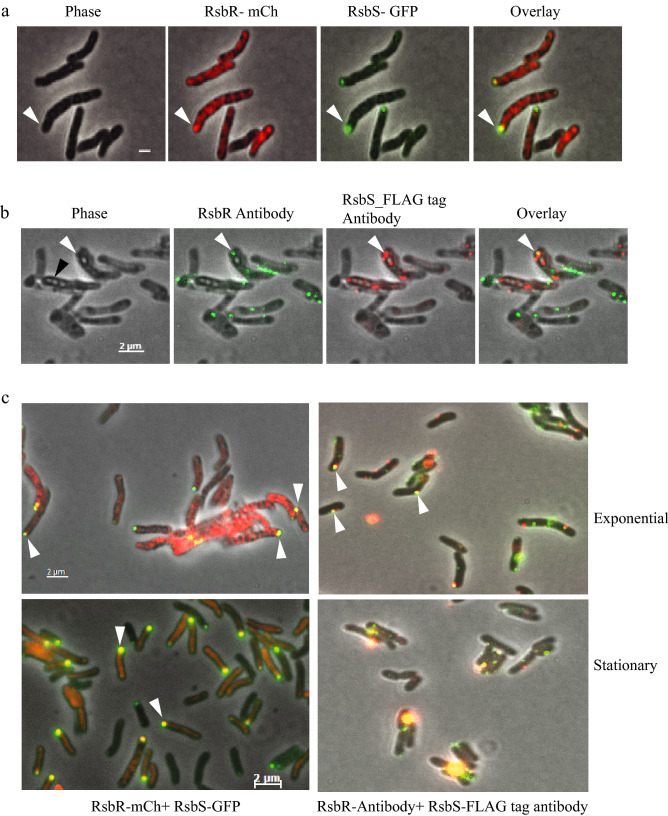
Co-localization of RsbR and RsbS in *Mmar*^T^ cells. (**a**) Shows the individual fields of RsbR-mCh, RsbS-GFP and an overlay with yellow spots representing the co-localization in exponential phase cells of *Mmar*^pST2K-RsbR-mCherry-RsbS-GFP^, scale bar = 1 μm. (**b**) Co-localization of RsbR and RsbS in *Mmar*^pSTKiT-RsbS-FLAG^ by immunofluorescence using antibodies against RsbR (secondary antibody tagged with Fluorescein—green) and against FLAG for RsbS (secondary antibody tagged with Alexafluor594—red). Yellow spots represent the RsbS-RsbR co-localization. (**c**) Shows co-localization of RsbR-RsbS (yellow spots indicated by white arrows) by fusion (first column) and immunofluorescence (second column) at exponential phase (top panel) and stationary phase (bottom panel).

RsbR-mCherry and RsbS-GFP fusion proteins were co-expressed from a single vector pST2K-RsbR:mCherry-RsbS:GFP (Table S2) and the locations of the fluorescent foci in live *Mmar*^T^ cells were examined. It should be noted that the fusion proteins RsbR-mCherry and RsbS_wt_-GFP (or when just one of the proteins were tagged) interact almost as well as the native proteins do (see Figs. S4 and S7a). We also examined co-localization of native RsbR and RsbS-FLAG proteins using antibodies raised against RsbR and the FLAG peptide (see above) and secondary antibodies conjugated with "Fluorescein (green)" and "Alexa-fluor 594 (red)" for RsbR and RsbS-FLAG, respectively. Irrespective of whether we studied fusion or native proteins, detection of yellow foci would indicate co-localization. The yellow spots with red and green foci at the same site but none in the phase image were taken as co-localized RsbR-RsbS complexes. Figure 5 shows the fluorescence microscopy images of co-localization of dye-tagged proteins in live cells (Fig. 5a) as well as that of immunofluorescence from fixed cells (Fig. 5b). Panels from left to right show the same fields in phase and with filters to reveal RsbR, RsbS and their overlap inside the bacterial cells. At this stage of growth, the exponential cells (2 days old) frequently showed refractile compartments similar to pre-spore structures (black arrow head; Fig. 5; these refractile compartments persisted in all images and were not counted to represent fluorescent foci). In a similar analysis with cells in stationary phase (cells collected ten days after inoculation) the frequency of yellow foci appeared to increase significantly (cf. Figure 5c and Table S8 for comparison between exponential and stationary phases). Furthermore, the yellow foci seemed stronger in intensity in stationary phase (Fig. 5c). Cells with the RsbR-mCherry fusion showed a high level of diffused fluorescence from free mCherry not seen in those with RsbS-GFP fusion or in cells stained with anti-RsbR antibody (discussed above). The red fluorescence from free mCherry were seen unaltered in the overlay images (rightmost panel in Fig. 5a). The yellow foci, representing RsbR-RsbS co-localized complex, seemed to occur at poles more frequently in stationary phase cells.

Cells with unipolar, bipolar and scattered distribution of foci were counted for RsbR, RsbS and RsbR-RsbS co-localized complexes and relative frequencies were estimated (Table S8). A summarized version of the proportions of polar and non-polar positions of the individual proteins and their complexes based on immunofluorescence data is given in Table S9a and b. Stationary phase cells showed a two-fold higher frequency of occupation at the poles compared to those in exponential phase while non-polar distribution of foci was reduced by more than half. Almost two- to three-fold higher proportion of RsbR and RsbS foci became part of the complex under the stationary phase.

## Discussion

The stress response in *B. subtilis* is administered by a multiprotein complex RsbRST, which plays a key role in activation of σ^B^, the general stress response σ-factor. Elegant genetic and structural analyses and micrographic imaging have revealed the stoichiometry of RsbRST compelxes called stresssomes and their intracellular distribution in *B. subtilis* during normal growth and under stress^14–18^. Previously, we have demonstrated that the RsbRST module is also present in *Mmar*, as well as in some other mycobacteria, and we have reported that this module is involved in the activation of the alternative σ-factor, σ^F^, in *Mmar*^18–22^. Here we expanded the bioinformatic data to include the majority of mycobacteria with known genome sequences and suggest that the RsbRST module is present in the majority of SGM while it is missing in the RGM with few exceptions. We also provide evidence that several RGM that lack RsbRST carry genes representing an alternative pathway involved in activating σ-factor(s), possibly σ^F^. This supports the presence of multiple genetic pathways involved in activating alternative σ-factors controlling the expression of different σ-mediated regulons needed to handle different stress conditions^19^. As in the *B. subtilis* system, our BACTH data suggested that the "STAS" domains in RsbR and RsbS are important for formation of the *M. marinum* RsbRS-complex^14,16^ (but note Co-IP data for RsbS, Table 1). Together, these data suggest that the genes as well as the interactions within the RsbRST module are conserved among the members of these two bacterial groups^22^ (see below). In addition, our results also indicate that the coiled-coil (CC) region in the RsbR C-terminus contribute to the establishment of this interaction. Lastly, using fluorescence microscopy, we have been able to demonstrate the intracellular locations of RsbR, RsbS and their complexes in live and fixed cells during vegetative growth and under stress (stationary phase) and show some differences in composition as well as in intracellular locations of stressosomes possibly affected by stress (stationary phase).

### Alternative pathways involved in activating σ-factors in mycobacteria

The alternative mycobacterial σ-factor σ^F^ is well conserved and it has a role in expression of genes in response to various stress conditions. The activity of σ^F^ in *Mycobacterium* spp. is controlled by an anti-σ factor, RsbW (also referred to as UsfX)^28^. The RsbRST-complex, RsbUW, RsbUVW and RsbV are likely to constitute the pathway that control the activity of RsbW and hence σ^F^ in *Mmar*^22^. Whether this is also the case for other mycobacteria that have these genes (Fig. S1) remains to be experimentally demonstrated but it is intuitively conceivable.

Analysis of available mycobacterial genomes suggested that homologs for *rsbW*, the gene encoding the anti-σ^F^ factor, exist in all these species. However, the RsbRST module was predicted to be present in SGM with few exceptions such as members of the TB-complex (Fig. S1). On the basis of the core phylogeny the SGM having the RsbRST module are likely to have received the corresponding genes after SGM diverged from RGM since the earliest linage, the *Mycobacterium chelonae* clade members lack RsbRST genes. Other pathway(s) that control σ^F^ activity in the species lacking RsbRST are yet to be identified. However, alternative σ^F^ activation routes have been characterized in several Firmicutes and in Actinomycetes such as *S. coelicolor*. The RsbKMY module has been suggested to be part of σ^F^ activation in *B. cereus* and *rsbK* and *rsbM* homologs have been recognized in *M. gilvum*^19,22^. We identified *rsbK* and *rsbM* homologs in 16 additional RGM strains (Figs. S1-S3). Comparison of the *rsbK* and *rsbM* gene synteny for these and two *M. gilvum* strains revealed other homologous genes upstream and downstream of *rsbK* and *rsbM*. One of these genes encodes a response regulator receiver modulated diguanylate cyclase/phosphodiesterase with a PAS/PAC sensor(s) (corresponding to Mflv_0999; Fig. S2). Whether it also is part of the "RsbK" route remains to be determined. Analysis of horizontally transferred genes in these *Mycobacterium* spp. further suggested that the *rsbK* gene probably originates from cyanobacteria [Supplementary information; for *rsbR*, *rsbS*, *rsbT* and *rsbM* our analysis did not provide any evidence supporting horizontal transfer of these genes into *Mmar* or in any of mycobacteria carrying RsbRST (Fig. S1)]. Given that we were unable to detect the presence of *rsbR*, *rsbS*, *rsbT*, *rsbK* and *rsbM* in *M. vaccae* might suggest yet a third route, "RsbPQX-route"^19^. However, we cannot exclude the possibility that the absence of the "RsbRST" and "RsbKMY" routes is due to that *M. vaccae* is a draft genome.

To conclude, the data supports the possibility that a route involving RsbK and RsbM does exist, at least in some mycobacteria. For other mycobacterial species that lack both RsbRST and RsbK (and RsbM) further studies are needed to identify the pathway(s) that influence the activation of the alternative σ-factor, σ^F^. In this context, we note that in *Mtb*, which lacks "RsbRST", "RsbKMY" and "RsbPQX", activation of σ^F^ occurs through a proteolytic pathway activated by the stress signal^29,30^.

### Intracellular localization of the *Mmar*^T^ RsbRST complex—Effects of stress

Intracellular locations of fluorescence-tagged RsbR and RsbS (mCherry and GFP, respectively) and their complexes were examined in live cells; the positions in their native forms were also seen using immunofluorescence microscopy. In both cases fluorescent foci were shown to be occupying cell poles as well as positions scattered in the cell. The difference seen in intracellular distribution of fluorescent foci with mCherry- and GFP-tagged proteins from those of the immuno-labeled native proteins in fixed cells called for some explanation. The large difference between the frequency of cells with fluorescent foci in RsbR-mCherry and RsbS-GFP-tagged cells (7% and 53%, respectively, of total with fluorescent foci; Table S5) arose from the overwhelming presence of free mCherry protein that contributed to diffused fluorescence not seen in Rsb-GFP-tagged cells (see Western Blot in Supplement Fig. S5; Fig. 2a,b). In contrast to the predominance of polar localization of RsbR-mCherry and RsbS-GFP foci in live cells, the native proteins show a more even distribution of immuno-labeled fluorescent foci between cell poles and cytoplasm. This difference could be attributed to the fluorescence-tagged RsbR and RsbS being almost twice as big as the native proteins. In addition, being expressed from multicopy plasmids, the tagged proteins could be in far excess compared to the native proteins, which were expressed from their single chromosomal loci. Furthermore, the fluorescent proteins at the ends of same size (RsbR and mCherry) and half size (RsbS and GFP) in excess of native proteins might interfere with RsbRST complex formation or remain free as RsbR-RsbR, RsbS-RsbS or RsbR-RsbS aggregates (Figs. S5 and S7b-d). All of these might contribute to the predominance of polar localization of RsbR and RsbS foci, in particular since excess proteins in aggregates are known to localize at cell poles^31^. The localization data for RsbS deletion proteins DN1, DN2, DC1 and 113N show that these protein fragments could localize at cell poles (Fig. 4a,b); the 113N-GFP construct might be too small to aggregate and form foci and GFP by itself does not form foci (Fig. 2a). Thus, the immunofluorescence foci for native proteins expressed from its chromosome in absence of stress might be a more reliable representation of the intracellular locations of stressosome proteins. But here too, almost one third of all foci are localized at cell poles and the rest are distributed over the cytoplasm. This is contrary to what has been observed in *B. subtilis* where immuno-localization experiments showed that RsbR as well as the RsbRST-complex form foci randomly distributed around the nucleoid in the cell without any preference for the cell-poles^16^. However, since only anti-RsbR-antibody was used to detect RsbRST complexes many of the immunofluorescent foci showed locations of RsbR, which may or may not be part of the RsbRST-complex. For the fluorescent foci to be counted as a RsbRST-complex, co-localization of RsbR and RsbS needed to be shown. As shown in our co-localization data, only 20–30% of RsbR/RsbS foci form part of "stressosome" complex during vegetative growth, which rises to 70–80% under stress (stationary phase; see Table S8). Both RsbR and RsbS can form oligomers, which might be visible as fluorescent foci in immuno-localization experiments. Even the *Mmar*^T^ RsbS, which based on our Co-IP data can interact with itself, and its deletion variants that do not interact with RsbR (but note that RsbS DN1 appears to interact with RsbR, Table 1), show up as fluorescent foci in significant numbers (see above and Fig. 4). We also emphasize that *Mmar*^T^ is equipped with only one RsbR gene while *B. subtilis* has several variants of RsbR, all of which might not form RsbRST-complex but might interact with the polyclonal antibody raised against one of the variants. As in the *B. subtilis* system the "STAS" domains^14,16^ of the RsbR (and possibly RsbS) are important for the formation of the *Mmar*^T^ RsbRST complex. Our data also indicate that the coiled-coil (CC) region in the RsbR C-terminus contribute to establishment of this interaction. Together, these data suggest that the genes as well as the physico-chemical interactions within the RsbRST module are conserved among the members of these two bacterial groups^22^. Our studies also indicate that the STAS-mediated RsbR-RsbS interaction plays a role in intracellular localization (see below).

The polar localization of "stressosome" components and "stressosome" itself could not be artefacts from the fused fluorescence protein alone since immuno-localization using anti-RsbR and anti-RsbS:FLAG antibodies also showed a significant proportion of polar localization of the native proteins and their complexes (see above and Fig. 5c). Thus, the polar locations of Rsb proteins and their complexes have to be considered significant and not artifacts from the experimental techniques. The shift in the relative frequencies of polar and non-polar foci in exponential (non-stress) and stationary (stressful) phases supports the non-trivial nature of the polar position of Rsb proteins' foci. We have no information about the structure of the RsbRST-complex in *Mmar*^T^ or its assembly process and its regulation. Therefore, we cannot exclude the possibility that the structure is different from that of the *B. subtilis* stressosome structure and that the localization and cellular environment might manifest this difference in the *Mmar*^T^ RsbRST structure. Noteworthy, the *Mmar*^T^ and *B. subtilis* RsbR N-termini shared poor homology^18^. Moreover, other mycobacterial proteins including the cell division protein Wag31 (the mycobacterial DivIVA homolog) and the proteins that constitute the secretion apparatus ESX-1 also localize at the cell pole^32–37^. For the DivIVA protein in *B. subtilis* and *S. coelicolor*, it has been suggested that the negative curvature at the poles has an important role for their preferential localization at the cell pole. It has also been discussed that the affinity of DivIVA for the phospholipid cardiolipin is a factor for polar localization since its concentration is higher at the poles in rod shaped bacteria^38–40^. Membrane-associated proteins can also be localized at the site where insertion of new peptidoglycan building blocks occurs (for a review see^41^). The mechanism behind the polar localization of RsbRST in *Mmar*^T^ as well as its structure remains to be deciphered but it is noteworthy that mycobacteria show polar growth similar to *S. coelicolor* whereas *B. subtilis* grows laterally^34,41–45^. Whether RsbR and RsbS are individually localized at cell poles or if it is a consequence of their assembly into the stressosome complex, which forms near the growing cell poles, cannot be ascertained from the experiments presented here since all the "stressosome" components were present in all the constructs while only a subpopulation was made visible with fluorescence tags or fluorescent antibody. However, the experiments with RsbS deletions that rendered them incapable of interaction with RsbR or RsbT showed that polar localization of RsbS did not depend on it being part of the stressosome complex; it might have a natural affinity for the cell pole (see Fig. 3). Thus, RsbS might be localized at the cell poles acting as a recruiting site for RsbR and other proteins for "stressosome" assembly. Increased frequency of polar localization under stress (Fig. 5b) is supportive of such a possibility.

## Methods

### Bacterial strains, media, growth conditions and gene constructions

The type strain *M. marinum* CCUG20998 (*Mmar*^T^) with and without different plasmids and the *Escherichia coli* Top 10 and BTH101 strains were grown as outlined in Supplementary information. The bacterial strains and plasmids are given in Tables S2–S4 in Supplementary information, and the primers used to generate these constructs are listed in Table S1.

Cloning, bacterial two-hybrid system and assessment of positive protein–protein interactions were performed as described in Supplementary information and have been published elsewhere^22^.

### Bioinformatics and phylogenetic analysis

The core gene phylogenetic tree was generated based on concatenated amino acid sequences of 56 core genes predicted to present in 244 mycobacteria for which genomes are available^4–7^ (Pettersson et al., to be published elsewhere). The amino acid sequences were aligned with MAFFT v 7.147b^46,47^, and the tree was constructed using the FastTree v2.1.7 tool^48,49^ with 1000 cycles of bootstrapping. The default settings were used, which infers approximately-maximum-likelihood phylogenetic tree using the Jones-Taylor-Thorton + CAT model for protein sequences. The phylogenetic tree was plotted using the interactive tree of life v3 (ITOLv3)^50^ and the gene synteny comparison we generated using GenoPlotR^51^.

The *Mmar rsbR* (MMAR_5182) and *rsbS* (MMAR_5183) sequences were retrieved from the NCBI database and analyzed with respect to the presence of "STAS" and coiled coil (CC) domains as outlined in Supplementary information.

The *rsb* genes constituting the σ^F^ activation pathway in different mycobacteria were identified as previously reported^16^ (see Supplementary information; Fig. S1). The *rsbK* and *rsbM* genes were analyzed as outlined in the text and in the Supplementary information (Fig. S1).

### Fluorescence microscopy, immunolocalization and western blot analysis

Fluorescence microscopy and immuno-localization of RsbR were performed as outlined below (see also Ref^32^). Briefly, cells were spotted on thin layers of 1% agarose in PBS formed on microscope slides. A Zeiss microscope (Axioplan 2) with a CCD camera linked to a computerized image analysis system was used for the micrographs. Images were analyzed using the program Axiovision 4.7. Filters used for green fluorescent protein, GFP, and mCherry fluorescence were HQ480/40 (480 ± 20 nm) and ET-Texas Red (550–630 nm), respectively. For immuno-localization studies, two-day-old (exponential phase) and 10 days old (stationary phases) cells were harvested from 7H10 plates, pelleted and re-suspended in 1X TBST (50 mM Tris–HCl pH 7.4, 150 mM NaCl, 0.1% Tween 80) containing antibodies (100X dilution) or pre-immune sera (for control). After incubation at room temperature for 1 h with agitation, the suspensions were washed twice with 1X TBST. All bacteria were then similarly incubated with fluorescently conjugated secondary antibodies [200X dilution; Fluorescein (green) or Alexa 594 (red)]. After washing three times with 1X TBST the bacteria were visualized as described above. Averages from two biological replicates were considered and number of cells considered/counted has been provided in the respective supplementary tables. For each condition, 10–15 frames per biological replicate. For microscopy data showing all channels of each image see Fig. S8.

The Western blot analysis was performed as described in methods, Supplementary information.

## Supplementary Information

Supplementary Information
