# Supplementary material for: Intracellular localization of the mycobacterial stressosome complex
Source: Sci Rep. 2021 May 12;11:10060. doi: 10.1038/s41598-021-89069-8 (PMC8115616; doi:10.1038/s41598-021-89069-8)

Supplementary information

Intracellular localization of mycobacterial stressosome complex: Role of STAS domain in protein-protein interactions

Malavika Ramesh[1], Ram Gopal Nitharwal[1,2], Phani Rama Krishna Behra[1], B.M. Fredrik Pettersson[1], Santanu Dasgupta[1] and Leif A. Kirsebom[1,*]

Department of Cell and Molecular Biology, Biomedical Centre, Uppsala University, Sweden.

[1]Department of Cell and Molecular Biology,
Biomedical Centre, Box 596, Uppsala University,
SE-751 24 Uppsala, Sweden
Tel: +46 18 471 4068; fax: +46 18 53 03 96
E-mail: leif.kirsebom@icm.uu.se

[2]Present address:
Department of Biotechnology,
Central University of Haryana,
Mahendergarh, 123031,
India

*Corresponding author Leif.Kirsebom@icm.uu.se

Supplementary Methods: Bioinformatics, Horizontally transferred genes, Bacterial strains, media and growth conditions, Gene constructions, Construction of rsbR and rsbS deletions and bacterial two-hybrid (BACTH) assay, Immunolocalization, and Western blot analysis.

Supplementary Table S1: Compilation of the different primers used in this study.

Supplementary Table S2: Lists the various plasmid constructs used in this study.

Supplementary Table S3: Compilation of the strains used in this study.

Supplementary Table S4: Compilation of the strains used for the two hybrid experiments.

Supplementary Table S5: RsbR and RsbS localisation in the cell using fusion proteins.
The average percentage of cells with foci and with dispersed fluorescence of RsbR-mCherry and RsbS-GFP when viewed under the confocal microscope (100x). The different positions of RsbR and RsbS in the cell with respect to total cells, with foci (not considering cells with dispersed fluorescence), is represented in brackets.

Supplementary Table S6: Cellular localization of RsbR and RsbS using Immuno-fluorescence.
The average percentage of cells with foci and with dispersed fluorescence of RsbR and RsbS were determined by Immuno-fluorescence using antibodies raised against RsbR and FLAG peptide (for RsbS). The different positions of RsbR and RsbS in the cell with respect to total cells, with foci (not considering cells with dispersed fluorescence), is represented in brackets.

Supplementary Table S7: Localization of RsbS in RsbS deletion mutants.

The average percentage of cells with foci and with dispersed fluorescence of RsbS deletions mutants were determined by GFP fluorescence when viewed under the confocal microscope (100x). The different positions in *Mmar*^{T} cells with respect to total cells, with foci (not considering cells with dispersed fluorescence), is represented in brackets.

Supplementary Table S8: Co-localization data using Immuno-fluorescence.

The average percentage of cells with foci of co-localization of RsbR and RsbS based on immune-fluorescence, where yellow foci represent co-localization.

Supplementary Table S9:

(a) Small and big yellow foci distribution.

The average percentage of cells with small and big yellow foci representing co-localization of RsbR and RsbS by immune-fluorescence. The different positions in the cell with respect to the total number of foci is represented in brackets.

(b) The percentage of total cells with yellow foci and the fraction of total cells having only 1, only 2 or >2 yellow foci.

Supplementary Methods

Bioinformatics

The *M. marinum* RsbR (MMAR_5182) sequence was retrieved from NCBI and subjected to the Expasy 'coils' tool for prediction of coiled coil regions (http://embnet.vital-it.ch/software/COILS_form.html). The probability score of 0.05 or above (on 14 amino-acids window) was considered to represent coiled coil (CC) conformation. It predicted five "CC" domains (1: 30 – 43; 2: 83 – 96; 3: 123 – 136; 4: 159 – 173; 5: 275 – 289) with "3" and "5" having low probability score of 0.008 and 0.039, respectively (an additional CC-domain with a low score = 0.033 was also predicted within the STAS domain, amino acids 198-212). Noteworthy, using the Simple Modular Architecture Research Tool (SMART, http://smart.embl-heidelberg.de/; Letunic and Bork, 2018)[50] did not predict an overlap between the RsbR "STAS" domain and CC-domain 5 (see main text).

Prediction of the "STAS" domain in RsbR was done using the Expasy 'prosite' tool (http://prosite.expasy.org). The RsbR showed the presence of a "STAS" domain (amino acids 174 – 285) with a score of 17.8. Similarly, RsbS (MMAR_5183) showed a "STAS" domain from 1-112 amino acids with a score of 15.62.

To identify *rsb* genes (RsbRST-module, RsbKM-module and RsbPQ related gene homologs) we performed the reciprocal blast hit method using the *Mmar*^{T} *rsb* genes (Pettersson et al, 2013)[22] and *Mycobacterium gilvum rsb* genes (de Been et al, 2011)[19] as input query sequences and targeted the 244 mycobacterial genomes. On the basis of the GFF annotation files (Seemann *et al.*, 2014)[52], Rsb-protein-coding related genes were extracted based on text search using the gene product/symbol as criteria. The *rsb* genes were considered to be present if the BLASTP results with the reciprocal blast hit had an e-value of at least 1e-03, a minimum protein percentage identity of 45% and a protein coverage >70% and GFF annotation along with the conserved gene synteny/ organization, *i.e.* similar to the *Mmar* M

strain (manually cross-checked) *rsb* gene synteny and also by considering the presence of conserved domains. Similarly, the *rsbK* and *rsbM* (*cheR* in *B. cereus*) genes were identified based on homology, domain architecture and gene synteny (de Been *et al*., 2011)[19]. The domain representation of RsbK protein was based on using the SMART tool (see above). NCBI gene identities for *rsbK* and *rsbM* in *M. gilvum* correspond to Mflv_1000 and Mflv_1001, respectively. The gene synteny analysis plot was generated using the genoPlotR tool (Guy *et al*., 2010)[51].

Horizontally transferred genes

Horizontally transferred *rsb* genes were identified using the HGTector 0.2.2 software (Zhu et al., 2014)[53]. HGTector uses the combination of the BLASTp approach and the taxonomy search for predicting putative horizontally transferred genes. The *rsb* gene sequences were subjected to BLASTp search analysis using the DIAMOND v 0.9.10 tool (Buchfink *et al*., 2015; Boratyn *et al*., 2013)[54,55] and the NCBI database (downloaded from the HGTector source v2017.6.30; Buchfink et al., 2015)[54]. The settings we used were: e-value = 0.0001, percentage identity >40% and query coverage >60%. For the taxonomy search (using HGTector) we used three parameters, "self" group, "close" group, and "distal" group hierarchical classification to predict putative horizontal genes where "self = Mycobacteria - species name", and "close = *Corynebacteriales*" (as of Feb 2018, NCBI taxonomy; Sayers et al. 2009)[56]. The "distal group" = all other organisms except the "self" and "close" groups (Zhu *et al*. 2014)[53].

Bacterial strains, media and growth conditions

The *Mmar*^{T} strain with and without different plasmids was grown on 7H10 agar plates or in 7H9 broth supplemented with 0.5% glycerol, 10% OADC (oleic acid albumin dextrose

complex) and 0.05% Tween 80. Antibiotic supplements were kanamycin (25 μg/ml), hygromycin (100 μg/ml) or both wherever needed.

Escherichia coli Top10 cells were grown on either LA agar plates supplemented with kanamycin (50 μg/ml), ampicillin (100 μg/ml) or hygromycin B (100 μg/ml) depending on the selection or in LB liquid media supplemented with one or more of the antibiotics mentioned above (for hygromycin B selection, the NaCl concentration was reduced to 5 g/L). The *E. coli* strain BTH101 (Str^R), used for bacterial two hybrid assays, was maintained on LA agar plates supplemented with 100 μg/ml streptomycin.

Gene constructions

All the primers required to generate the constructs are listed in Table S1. *Mmar*^{T} genomic DNA was used for PCR amplification (Phusion DNA polymerase, Thermo Scientific) of the *rsbR* and *rsbS* genes. The *rsbR* fused with the mCherry gene was generated in the *E. coli/ Mycobacterium* shuttle vector, pBS401 (Singh *et al*., 2013)[45]. First the *rsbR* gene (MMAR_5182) was PCR amplified (primers 1 and 2) and cloned between the *Afl*II and *Nde*I restriction sites generating pBS401-RsbR. The mCherry gene was PCR amplified using primers 3 and 4 and pRSET-B-mCherry as template (Shaner, 2004)[57]. The PCR product was cloned between the *Nde*I and *Spe*I sites in pBS401-RsbR. The resulting construct is referred to as pBS401-RsbR-mCherry.

Due to limitation of suitable restriction sites in the pST-2K vector, the RsbS-GFP fusion cassette was first generated in pBS401 (using the primer pairs 5 and 6, and 7 and 8) in a similar way as described for the construction of pBS401-RsbR-mCherry. This construct was referred to as pBS401-RsbS-GFP. The RsbS-GFP fusion cassette was PCR amplified (using primers 9 and 10) and cloned into the pST-2K vector (Parikh *et al*., 2013)[58] between the *EcoR*I and *Hind*III sites in the MC2 region yielding pST2K-RsbS-GFP.

For the co-localization studies a construct expressing both RsbR-mCherry and RsbS-GFP fusion proteins from a single plasmid (pST2K-RsbR-mCherry-RsbS-GFP) was generated. The RsbR-mCherry cassette was PCR amplified using pBS401-RsbR-mCherry as template and the primer pair 11 and 12. This PCR product was cloned between the *BamH*I and *Xba*I sites in the MC1 region in the pST2K-RsbS-GFP construct.

The RsbS-GFP N-terminal deletions ($RsbS_{DN1}$-GFP, $RsbS_{DN2}$-GFP, and $RsbS_{113N}$-GFP) were PCR amplified from pST2K-RsbS-GFP using the following primer pairs; DN1 (13 and 10), DN2 (14 and 10) and 113N (15 and 10). These PCR products were cloned between the *EcoR*I and *Hind*III sites in the pST2K vector as described above. For the generation of pST2K-$RsbS_{DC1}$-GFP the pST2K-RsbS-GFP constructs was directly PCR amplified using the phosphorylated primers (16 and 17). The PCR product was *DpnI* digested, circularized by ligation, transformed into *E. coli* Top10 cells and selected on LA agar plates supplemented with 50 µg/ml kanamycin.

One µg of plasmid DNA with the different constructs were electroporated in $Mmar^T$ cells at 2.5 kV, 25 µF and 1000 Ω using pre-chilled 0.2 cm gap cuvettes. The electroporation mixture (200 µl) was removed immediately after electroporation, mixed with 2 ml of 7H9 medium supplemented with 10% OADC and kept at 30°C overnight; positive transformants were selected on 7H10 plates containing appropriate antibiotics.

Construction of *rsbR* and *rsbS* deletions and bacterial two-hybrid (BACTH) assay

Various deletions of RsbR and RsbS were made in pKT25 and pUT18C vectors, respectively, as described in the main text and elsewhere (Pettersson *et al*., 2013; Karimova *et al*., 1998; Ladant & Karimova, 2000; Euromedex)[22,59,60]. For the BACTH interaction studies we used the previously cloned *rsbR* (pKT25 and pUT18), *rsbS* (pUT18C) and *rsbT* (pKT25 and pUT18) in different vectors (Petterson *et al*., 2013)[22]. The pKT25-RsbR construct was used as a

template to generate the RsbR N- and C-terminal deletion mutants. The primer pairs that were used to construct RsbR DN1-5 and DC1-6 are listed in Table S1: $RsbR_{DN1}$ primers 18 and 19; $RsbR_{DN2}$ primers 18 and 20; $RsbR_{DN3}$ primers 18 and 21; $RsbR_{DN4}$ primers 18 and 22; $RsbR_{DN5}$ primers 18 and 23; $RsbR_{DC1}$ primers 24 and 25; $RsbR_{DC3}$ primers 24 and 26; $RsbR_{DC4}$ primers 24 and 27; $RsbR_{DC5}$ primers 24 and 28; $RsbR_{DC6}$ primers 24 and 29. The PCR products were digested with *Dpn*I and *Xba*I (N-terminal deletions), and *Dpn*I and *Kpn*I (C-terminal deletions). The PCR fragments were circularized using T4 DNA ligase and transformed in *E. coli* Top 10 cells. Transformants were selected on LA agar plates supplemented with 50 µg/ml kanamycin.

The *rsbS* deletion mutants were generated following a similar protocol using the pUT18C-RsbS (Pettersson *et al*., 2013)[22] construct as template. The following primer pairs were used (see Table S1): $RsbS_{DN1}$ primers 30 and 31; $RsbS_{DN2}$ primers 30 and 32; $RsbS_{DC1}$ primers 33 and 34; $RsbS_{DC2}$ primers 33 and 35. The PCR fragments were digested with *Dpn*I and *Xba*I (N-terminal deletions) and *Dpn*I and *Kpn*I (C-terminal deletions). After ligation, the different constructs were transformed into *E. coli* Top10 cells. Transformants were selected on LA agar plates supplemented with 100 µg/ml ampicillin. The different constructs were confirmed by DNA sequencing.

The resulting plasmids were transformed pairwise into the *E. coli* strain BTH101 (Str^R) and streaked on LA-plates supplemented with 50 µg/ml kanamycin, 100 µg/ml ampicillin, and 100 µg/ml streptomycin. Colonies were re-streaked and plated on LA-plates containing 50 µg/ml kanamycin, 100 µg/ml ampicillin, 40 µg/ml X-Gal, and 0.5 mM IPTG according to the manufacturer's protocol; formation of blue colonies was scored as a positive interaction.

Construction of the co-immunoprecipitation constructs

The co-immunoprecipitation constructs were cloned pairwise in the pET-28a(+) plasmid, one protein with a 6xHis-tag and the other protein with a FLAG-tag. For the majority of the constructs, the plasmid encoded 6xHis tag was used for making N-terminal 6xHis tag fusions by cloning the protein genes in frame at the *Nde*I site. The C-terminal ends of the His-tagged protein ORFs were cloned into the *Sac*I site. Primer pairs to construct: His-RsbR (pairs, 39 and 40), His-RsbRDC1 (pairs, 39 and 41), His-RsbRDN4 (pairs, 51 and 40), His-RsbRDN5 (pairs, 52 and 40) and His-RsbT (pairs, 53 and 54). All primers are listed in Table S1.

For the RsbS-His construct (with a C-terminal His-tag), the *Nco*I site, which is located upstream of the *Nde*I site but downstream of the translation initiation region in pET-28a(+), was used to remove the plasmid-encoded 6xHis-tag (located between the *Nco*I and *Nde*I sites).

The C-terminal 6xHis-tag was introduced with primer 56, which together with primer 55 was used to clone the RsbS-His between the *Nco*I and *Sac*I sites.

The FLAG-tag was introduced by PCR and the FLAG-peptide tagged protein constructs were cloned downstream of the 6xHis-tagged proteins between the *Sac*I and *Hind*III sites in pET-28a(+). The forward primers introduced a copy of the translation initiation region from the plasmid encoded ORF expressing the 6xHis-tag to ensure a similar translation efficiency of both the His-tagged and the FLAG-tagged proteins. Primer pairs to construct: the FLAG-RsbR (pairs 42 and 43), the FLAG-RsbRDN5 (pairs, 45 and 43), the RsbS-FLAG (pairs, 46 and 47), the RsbSDN1-FLAG (pairs 48 and 47) and the RsbT-FLAG (pairs, 49 and 50).

The double constructs were generated: i) cloning either the FLAG-tagged or the 6xHis-tagged protein into pET-28a(+), ii) purifying and cutting the resulting plasmid with *Nde*I (*Nco*I for RsbS-His) and *Sac*I (when FLAG-tagged proteins were cloned first) or *Sac*I and *Hind*III (when 6xHis-tagged proteins were cloned first), and iii) cutting the PCR-product of

the second construct with the same enzymes followed by ligation. All generated constructs were verified by sequencing and are listed in Table S2b-d.

Immunolocalization of RsbR

Immunolocalization of RsbR was performed as described elsewhere for *Mycobacterium* spp. (Carlsson *et al*., 2009)[32]. Briefly, one-week-old cells were harvested from 7H10 plates, pelleted and suspended in 1X TBST (20 mM Tris-HCl pH 7.4, 137 mM NaCl, 0.05% Tween 80) containing RsbR antibodies (200X dilution). After incubation at room temperature for 1 h with agitation, the suspensions were washed twice with 1X TBST. All bacteria were then similarly incubated with fluorescently conjugated secondary antibodies [FITC (green) or Alexa 594 (red)]. After washing three times with 1X TBST the bacteria were placed on a thin 1% agarose pad on slides and covered with a coverslip. The visualization of bacteria and detection of fluorescence were done with a fluorescence microscope (see above).

Western blot analysis

Mmar^{T} cells harbouring appropriate constructs as shown in Fig S4 (cultivated on 7H10 solid media for one week) were washed in TBS buffer 2-3 times and dissolved in lysis buffer (1X TBS, 2% Triton X100, 1 mM EDTA, 200 mM DTT, 5M urea). The cell suspension was transferred to centrifuge tubes containing 0.1 mm zirconium beads. The cells were disrupted in a bead beater for 30 seconds with 30 seconds resting on ice (four cycles). The lysates were cleared by centrifugation at 13000 rpm for 5 minutes. Equal volume of 2X SDS loading buffer was added to the cleared lysates and the mixture was boiled at 95ºC for 5 minutes. The samples were loaded and resolved on 10% SDS-PAGE along with molecular weight standards. The proteins were transferred to PVDF membranes and blocked by 2.5 % BSA. The mCherry and GFP primary antibodies were used for detection of RsbR-mCherry and

RsbS-GFP, respectively. The appropriate secondary antibodies tagged with horseradish peroxidase (HRP) were used. The chemiluminescence was developed by ECL reagent (Clarity, BioRad) and the signal was detected by a CCD camera attached to a chemiDoc system (BioRad). The Image lab software (BioRad) was used for image optimization and capture. The quantitation of the individual bands was done by ImageJ software.

Co-immunoprecipitation (Co-IP) and Co-purification of interacting proteins

The RsbR, RsbS, and RsbT genes and deletion constructs (see Fig S4e-f) were cloned pairwise, with added 6xHis-tags or FLAG-peptide tags, into the expression vector pET-28a(+) as outlined above (see Table S1 for primers). The proteins were cloned in such a way that both proteins in the pairs were co-expressed from the single T7-promoter of the pET-28a(+) plasmid. The plasmids were transformed into *Escherichia coli* BL21 DE3 cells. Cultures were initially inoculated with a single colony and grown in 20 ml Terrific Broth (TB) supplemented with 50 µg/ml Kanamycin at 37°C overnight, the culture was diluted 100-fold in the same media and 200 ml cultures were grown at 37°C until OD_{600} ~0.5. IPTG (isopropyl β-D-1-thiogalactopyranoside) was added to a final concentration of 1 mM to induce expression of the constructs (through induction of T7 RNA polymerase). The cultures were grown for an additional 24 hours at room temperature, harvested by centrifugation, and stored in 15 ml aliquots at -20°C. The cells were re-suspended in 1 ml of TBS (<u>T</u>ris <u>b</u>uffered <u>s</u>aline: 25 mM Tris, 137 mM NaCl, 5.0 mM KCl, 0.69 mM $CaCl_2$, 0.36 mM $MgCl_2$, 0.62 mM NaH_2PO_4, pH 7.4) per 5 ml of original culture volume and cell lysates were prepared according to the Pierce Pull-Down PolyHis Protein:Protein Interaction Kit following the steps for bait protein preparation.

To 1 ml of crude lysate 10 µl of rabbit anti-FLAG antibody (Invitrogen, PA1-984B) was added and the samples were incubated overnight at +4°C with gentle agitation. Protein-G

agarose beads (50 μl settled bead volume) were rinsed with 1 ml of Pierce Pull-Down PolyHis Protein:Protein Interaction Kit wash buffer and the lysate/antibody mixtures were transferred to the beads. The samples were incubated at +4°C for 30 min followed by 1.5 hr incubation at room temperature with constant gentle agitation. The protein-G/antibody/protein complexes were washed 5x for 2 min with wash buffer and 2 times with 1 x TE buffer (50 mM Tris, pH 7.9/ 1 mM EDTA) with 2 min pelleting of the complexes at 2000 x g between each wash. Finally, the proteins were eluted with 150 μl of elution buffer (50 mM Tris-HCl, pH 7.9; 150 mM NaCl; 0.1% NP40) at 65°C for 15 min. After saving the eluate, the beads were incubated for 5 min at 65°C with 1% SDS in 1 x TE buffer. The eluate from both procedures were stored separately at -20°C.

Analysis of co-immunoprecipitated proteins by Western blotting

The protein lysates and eluates (the eluates from elution 2 were used as preliminary experiments indicated that this harsher elution was needed to elute the proteins) were mixed with and equal volume of 2x Laemmli buffer and 17 μl aliquots were loaded on an Any-kD ready-made SDS-PAGE gel (BioRad) together with a PAGE-ruler size marker (Thermo-Fisher) and run (at 300V constant voltage) until the Bromophenol blue dye reached the bottom of the gel. The proteins were blotted to an Amersham Hybond low fluorescence PVDF blotting membrane (0.2 μm pore size) following the manufacturer's instructions. The membranes were blocked using 0.1% (v/v) Tween 20 and 5% (w/v) dried skimmed milk in TBS (see above) for 1 h. Primary antibodies targeting the FLAG-tag (PA1-984B; 1:500 dilution; from rabbit) and the 6xHis-tag (MA1-21315; 1:2000; from mouse; Thermo-Fisher) were added to the membranes in the blocking solution and incubation continued overnight at +4°C. The blots were washed in TBS containing 0.1% (v/v) Tween 20 (TBS-T) by incubation for 4x5 min. The blots were incubated with fluorescence conjugated secondary antibodies

targeting the primary anti-FLAG peptide (A32734, Alexa Fluor PLUS 680 goat anti-rabbit IgG; 1:10000 dilution; from goat) and the anti-6xHis (A32723, Alexa Fluor PLUS 488 goat anti-mouse IgG; 1:20000 dilution; from goat) antibodies (Thermo-Fisher) in blocking solution for 1 h. The blots were washed in TBS-T as above. All incubations were carried out at room temperature except where noted. Fluorescence was detected using a ChemiDoc MP imager (BioRad) and the data presented are the result of one replicate.

References supporting information (see also main text)

52. Seemann, T. Prokka: rapid prokaryotic genome annotation. *Bioinformatics* **30**, 2068–2069 (2014).

53. Zhu, Q., Kosoy, M. & Dittmar, K. HGTector: an automated method facilitating genome-wide 152 discovery of putative horizontal gene transfers. *BMC Genomics* **15**, 717 (2014).

54. Buchfink, B., Xie, C. & Huson, D.H. Fast and sensitive protein alignment using DIAMOND. *Nat Meth* **12**, 59–60 (2015).

55. Boratyn G.M., Camacho, C., Cooper, P.S., Coulouris, G., Fong, A., Ma, N., *et al*. BLAST: a more efficient report with usability improvements. *Nucleic Acids Res.* **41**, W29–33 (2013).

56. Sayers, E.W. et al. Database resources of the National Center for Biotechnology Information. *Nucleic Acids Res* **37**, D5-15 (2009).

57. Shaner, N.C., Campbell, R.E., Steinbach, P.A., Giepmans, B.N., Palmer, A.E. & Tsien, R.Y. Improved monomeric, orange and yellow fluorescent proteins derieved from *Discosoma sp.* Red fluorescent protein. *Nat Biotechnol* **22**, 1567-1572 (2004).

58. Parikh, A., Kumar, D., Chawla, Y., Kurthoti, K., Khan, S., Varshney, U. & Nandicoori, V. Development of a new generation of vectors for gene expression, gene replacement, and protein-protein interaction studies in mycobacteria. *Appl Environ Microbiol* **79**, 1718-1729 (2013).

59. Karimova, G., Pidoux, J., Ullmann, A. & Ladant, D. A bacterial two-hybrid system based on a reconstituted signal transduction pathway. *Proc Natl Acad Sci USA* **95**, 5752–5756 (1998).

60. Ladant, D. & Karimova, G. Genetic systems for analyzing protein-protein interactions in bacteria. *Res Microbiol* **151**,711–720 (2000).

61. Chen, L-C., Chen, J-C., Shu, J-C., Chen, C-Y., Chen, S-C., Chen, S-H., Lin, C-Y., Lu, C-Y. & Chen, C-C. Interplay of RsbM and RsbK controls the σ^B activity of *Bacillus cereus*. *Environ Microbiol* **14**, 2789-2799 (2012).

Table S1 Compilation of the different primers used in the present study

No.	Primer sequence (5'-3')
1.	TTTTCTTAAGTGAGTACCACCGAAGGGGTGTATGTGAGTGCGCTCGCCGATGTCAGATTCGCCG
2.	CGCATATGGTCGCCGGTGCGAGTG
3.	CGCATATGATGGTGAGCAAGGGCG
4.	TTACTAGTTCGAGCGCCTCGCGTGAGCGAGGCGCTCGATCACTTGTACAGCTCGTCCATGCC
5.	TTTTCTTAAGTGAGTACCACCGAAGGGGTGTATGTGAGTGCGCTCGCCGATGCCAGTACCGATCTTGAAACA
6.	TTTTCATATGTCTAGATCCGCCACCGTCGGG
7.	TTTTCATATGATGAGTAAAGGAGAAGAACTTTTC
8.	TTACTAGTTCGAGCGCCTCGCGTGAGCGAGGCGCTCGATCATTTGTATAGTTCATCCATGCCAT
9.	TTTTGAATTCATGCCAGTACCGATCTTG
10.	TTTTAAGCTTTTTGTATAGTTCATCCATG
11.	TTTTGGATCCATGTCAGATTCGCCGTCC
12.	TTAATCTAGATTACTTGTACAGCTCGTCCA
13.	TTTTGAATTCATGATTGTGGTCGACGTGAC
14.	TTTTGAATTCATGTTTTCAATGGTCCAATTGGGCCTGACCTTC
15.	TTTTGAATTCAACAAGCAACTGGGACTACG
16.	TCTAGACATATGATGAGTAAAGGAGAAG
17.	CGCGACTTCTGGCTGCAGGCCCACGA
18.	CTCGAATTCGTGATTCCTCCGGATCCTGG
19.	CCAGGATCCGGAGGAATCACGAATTCGAG
20.	TTTACTCTAGAGTCGACCCTGCAGCCC
21.	TTTTACTCTAGAGAAGTGGGCCCGCAGCAT
22.	TTTTACTCTAGAGATCATTCCCAGGGGCGTG
23.	TTTTACTCTAGAGCTGGACGCTTACGAACC
24.	TTTTACTCTAGAGTCCACGCCCGTGCTGCA
25.	TTTTACTCTAGAGGGCGATTTACAAGGGGGAAT
26.	TTTCGGTACCTAAGTAACTAAGAATTCGGCCG
27.	TTTCGGTACCCACGGCATTCATCTTCGAAAG
28.	TTTCGGTACCTAACTCGGCTGAGCATCTCGAA
29.	TTTCGGTACCGCGTTCGGAGAGATCACGT
30.	TTTCGGTACCTCGCGCAACGCGGTT
31.	TTTCGGTACCCAGCTGCGGCAGCAAAC
32.	TTTTCTAGAGTCGACCTGCAGTGGCG
33.	TTTTTCTAGAGATTGTGGTCGACGTGAC
34.	TTTTTCTAGAGTTTTCAATGGTCCAATTGGGCCTGACCTTC
35.	TTTTGGTACCGAGCTCGAATTCATCGAT
36.	TTTTGGTACCCGCGACTTCTGGCTGCAGGCCCACGA
37.	TTTTGGTACCGCCCTGGGCGCGAAAGCGGCTGAC
38.	TTTTGAATTCATGAGTAAAGGAGAAGAACTTTTC
39	TTTTCATATGTCAGATTCGCCGTCCGTC
40	TTTGAGCTCCTAGTCGCCGGTGCGAGTGACTGC
41	TTTGAGCTCCTACACGGCATTCATCTTCGAAAG
42	TTTGAGCTCAAGAAGGAGATATACCATGGACTACAAGGACGACGACGACAAGATGTCAGATTCGCCGTCCGTC
43	TTTAAGCTTCTAGTCGCCGGTGCGAGTGACTGC
44	TTTAAGCTTCTACACGGCATTCATCTTCGAAAG
45	TTTGAGCTCAAGAAGGAGATATACCATGGACTACAAGGACGACGACGACAAGGTGGGCGATTTACAAGGGGGTAT
46	TTTGAGCTCAAGAAGGAGATATACCATGCCAGTACCGATCTTGAAAC
47	TTTAAGCTTTTACTTGTCGTCGTCGTCCTTGTAGTCTCCGCCACCGTCGGGCCCGATC
48	TTTGAGCTCAAGAAGGAGATATACCATGATTGTGGTCGACGTGACCG
49	TTTGAGCTCAAGAAGGAGATATACCATGGTGGTCGGGATCAACCATC
50	TTTAAGCTTTTACTTGTCGTCGTCGTCCTTGTAGTCCGCACGAGGCGGAACCCATTTC
51	TTTCATATGTCCACGCCCGTGCTGCAGGTG
52	TTTCATATGGGCGATTTACAAGGGGGTAT
53	TTTTCATATGGTGGTCGGGATCAACCATC
54	TTTGAGCTCCTACGCACGAGGCGGAACCCATTTC
55	TTTCCATGGGCATGCCAGTACCGATCTTGAAAC
56	TTTGAGCTCTTAGCTGCTGTGATGATGATGATGATGGCTGCTGCCCATTCCGCCACCGTCGGGCCCGATC

Table S2 Plasmid constructs used in this study

a

No.	Plasmid/Construct name	Source
	Constructs for localization studies	
1.	pBS401	Singh, B., et al. 2013
2.	pBS401-RsbR-mCherry	This study
3.	pBS401-mCherry	This study
4.	pST-2K	Parikh, A., et al. 2013
5.	pST2K-GFP	This study
6.	pST2K-RsbS-GFP	This study
7.	pST2K-RsbR-mCherry-RsbS-GFP	This study
8.	pST2K-RsbSDN1-GFP	This study
9.	pST2K-RsbSDN2-GFP	This study
10.	pST2K-RsbS113N-GFP	This study
11.	pST2K-RsbSDC1-GFP	This study
12.	pSTKiT-RsbS-FLAG	This study
	Constructs for bacterial two hybrid studies	
13.	pKT25-RsbR	Pettersson et al., 2013
14.	pUT18-RsbR	Pettersson et al., 2013
15.	pKT25-RsbR DN1	This study
16.	pKT25-RsbR DN2	This study
17.	pKT25-RsbR DN3	This study
18.	pKT25-RsbR DN4	This study
19.	pKT25-RsbR DN5	This study
20.	pKT25-RsbR DC1	This study
21.	pKT25-RsbR DC3	This study
22.	pKT25-RsbR DC4	This study
23.	pKT25-RsbR DC5	This study
24.	pKT25-RsbR DC6	This study
25.	pUT18C-RsbS	Pettersson et al., 2013
26.	pUT18C-RsbSDN1	This study
27.	pUT18C-RsbSDN2	This study
28.	pUT18C-RsbSDC1	This study
29.	pUT18C-RsbSDC2	This study
30.	pUT18-RsbT	Pettersson et al., 2013
31.	pKT25-RsbT	Pettersson et al., 2013
32.	pUT18	Euromedex
33.	pUT18C	Euromedex
34.	pKT25	Euromedex
35.	pKT25-ZIP	Euromedex
36.	pUT18C-ZIP	Euromedex

b

	PCR products			
1	A+B	pET28a(+)	His-R-Stop	34716,52 Daltons
2	A+C	pET28a(+)	His-RDC1-Stop	32314,05 Daltons
3	D+E	pET28a(+)	FLAG-R-Stop	33679,38 Daltons
4	D+F	pET28a(+)	FLAG-RDC1-Stop	31276,91 Daltons
5	G+E	pET28a(+)	FLAG-RDN5-Stop	3645,72 Daltons
6	H+I	pET28a(+)	S-FLAG-Stop	14910,31 Daltons
7	I+J	pET28a(+)	SDN1-FLAG-Stop	10212,15 Daltons
8	K+L	pET28a(+)	T-FLAG-Stop	15161,42 Daltons
9	B+M	pET28a(+)	His-RDN4-Stop	15460,94 Daltons
10	B+N	pET28a(+)	His-RDN5-Stop	4714,92 Daltons
11	O+P	pET28a(+)	His-T-Stop	16329,75 Daltons
12	Q+R	pET28a(+)	S-His-Stop	15463,00 Daltons

c

Coexpression Constructs				
		Rwt	Swt	Twt
	Rwt	1+3	1+6	1+8
Same as RDN4	RDN1	nd	nd	nd
Same as RDN4	RDN2	nd	nd	nd
Same as RDN4	RDN3	nd	nd	nd
	RDN4	3+9	6+9	8+9
	RDN5	1+5	6+10	8+10
	RDC1	nd	2+6	2+8
Same as RDC1	RDC3	nd	nd	nd
Same as RDC1	RDC4	nd	nd	nd
Same as RDC1	RDC5	nd	nd	nd
Same as RDN5	RDC6	nd	nd	nd
		Rwt	Swt	Twt
	Swt	1+6	6+12	6+11
	SDN1	1+7	7+12	7+11
Same as RDN1	SDN2	nd	nd	nd
Same as RDN1	SDC1	nd	nd	nd
Same as RDN1	SDC2	nd	nd	nd

d

Coexpression construct	
1+3	FLAG-R-His-R
3+9	FLAG-R-His-RDN4
1+5	FLAG-RDN5-His-R
1+6	S-FLAG-His-R
1+7	SDN1-FLAG-His-R
6+9	S-FLAG-His-RDN4
6+10	S-FLAG-His-RDN5
2+6	S-FLAG-His-RDC1
7+12	SDN1-FLAG-S-His
6+12	S-FLAG-S-His
7+11	SDN1-FLAG-His-T
6+11	S-FLAG-His-T
2+8	T-FLAG-His-RDC1
8+10	T-FLAG-His-RDN5
8+9	T-FLAG-His-RDN4
1+8	T-FLAG-His-R

Table S3 Strains used for localization studies

No.	Strain name	Relevant genotype	Source
1.	*Mmar*T	*M. marinum* CCUG 20998	Regular lab strain
2.	*Mmar*pBS401-mCherry	*M. marinum* CCUG 20998/pBS401-mCherry	This study
3.	*Mmar*pBS401-RsbR-mCherry	*M. marinum* CCUG 20998/pBS401-RsbR-mCherry	This study
4.	*Mmar*pST2K-GFP	*M. marinum* CCUG 20998/pST2K-GFP	This study
5.	*Mmar*pST2K-RsbS-GFP	*M. marinum* CCUG 20998/pST2K-RsbS-GFP	This study
6.	*Mmar*pST2K-RsbS-GFP-RsbR-mCherry	*M. marinum* CCUG 20998/pST2K-RsbR-mCherry-RsbS-GFP	This study
7.	*Mmar*pST2K-RsbS-DN1-GFP	*M. marinum* CCUG 20998/pST2K-RsbSDN1-GFP	This study
8.	*Mmar*pST2K-RsbS-DN2-GFP	*M. marinum* CCUG 20998/pST2K-RsbSDN2-GFP	This study
9.	*Mmar*pST2K-RsbS-113N-GFP	*M. marinum* CCUG 20998/pST2K-RsbS113N-GFP	This study
10.	*Mmar*pST2K-RsbS-DC1-GFP	*M. marinum* CCUG 20998/pST2K-RsbSDC1-GFP	This study
11.	*Mmar*pSTKiT-RsbS-FLAG	*M. marinum* CCUG 20998/pSTKiT-RsbS-FLAG	This study

Table S4 Strains used for bacterial two hybrid

No.	Strain name	Relevant genotype	Source
1	BTH101	Bacterial two hybrid *E. coli* strain	Euromedex
2	BTHRG1	BTH101/pUT18-RsbR/pKT25-RsbR	This study
3.	BTHRG2	BTH101/pUT18-RsbR/pKT25-RsbR	This study
4.	BTHRG3	BTH101/pUT18-RsbR/pKT25-RsbR DN1	This study
5.	BTHRG4	BTH101/pUT18-RsbR/pKT25-RsbR DN2	This study
6.	BTHRG5	BTH101/pUT18-RsbR/pKT25-RsbR DN3	This study
7.	BTHRG6	BTH101/pUT18-RsbR/pKT25-RsbR DN4	This study
8.	BTHRG7	BTH101/pUT18-RsbR/pKT25-RsbR DN5	This study
9.	BTHRG8	BTH101/pUT18-RsbR/pKT25-RsbR DC1	This study
10.	BTHRG9	BTH101/pUT18-RsbR/pKT25-RsbR DC3	This study
11.	BTHRG10	BTH101/pUT18-RsbR/pKT25-RsbR DC4	This study
12.	BTHRG11	BTH101/pUT18-RsbR/pKT25-RsbR DC5	This study
13.	BTHRG12	BTH101/pUT18-RsbR/pKT25-RsbR DC6	This study
14.	BTHRG13	BTH101/pUT18 /pKT25	This study
15.	BTHRG14	BTH101/pUT18C-RsbS/pKT25-RsbR	This study
16.	BTHRG15	BTH101/pUT18C-RsbS/pKT25-RsbR DN1	This study
17.	BTHRG16	BTH101/pUT18C-RsbS/pKT25-RsbR DN2	This study
18.	BTHRG17	BTH101/pUT18C-RsbS/pKT25-RsbR DN3	This study
19.	BTHRG18	BTH101/pUT18C-RsbS/pKT25-RsbR DN4	This study
20.	BTHRG19	BTH101/pUT18C-RsbS/pKT25-RsbR DN5	This study
21.	BTHRG20	BTH101/pUT18C-RsbS/pKT25-RsbR DC1	This study
22.	BTHRG21	BTH101/pUT18C-RsbS/pKT25-RsbR DC3	This study
23.	BTHRG22	BTH101/pUT18C-RsbS/pKT25-RsbR DC4	This study
24.	BTHRG23	BTH101/pUT18C-RsbS/pKT25-RsbR DC5	This study
25.	BTHRG24	BTH101/pUT18C-RsbS/pKT25-RsbR DC6	This study
26.	BTHRG25	BTH101/pUT18C- /pKT25	This study
27.	BTHRG26	BTH101/pUT18-RsbT/pKT25RsbR	This study
28.	BTHRG27	BTH101/pUT18-RsbT/pKT25RsbR DN1	This study
29.	BTHRG28	BTH101/pUT18-RsbT/pKT25RsbR DN2	This study
30.	BTHRG29	BTH101/pUT18-RsbT/pKT25RsbR DN3	This study
31.	BTHRG30	BTH101/pUT18-RsbT/pKT25RsbR DN4	This study
32.	BTHRG31	BTH101/pUT18-RsbT/pKT25RsbR DN5	This study
33.	BTHRG32	BTH101/pUT18-RsbT/pKT25RsbR DC1	This study
34.	BTHRG33	BTH101/pUT18-RsbT/pKT25RsbR DC3	This study
35.	BTHRG34	BTH101/pUT18-RsbT/pKT25RsbR DC4	This study
36.	BTHRG35	BTH101/pUT18-RsbT/pKT25RsbR DC5	This study
37.	BTHRG36	BTH101/pUT18-RsbT/pKT25RsbR DC6	This study
38.	BTHRG37	BTH101/pKT25-RsbR/ pUT18C-RsbSDN1	This study
39.	BTHRG38	BTH101/pKT25-RsbR/ pUT18C-RsbSDN2	This study
40.	BTHRG39	BTH101/pKT25-RsbR/ pUT18C-RsbSDC1	This study
41.	BTHRG40	BTH101/pKT25-RsbR/ pUT18C-RsbSDC2	This study
42.	BTHRG41	BTH101/pKT25-RsbT/ pUT18C-RsbSDN1	This study
43.	BTHRG42	BTH101/pKT25-RsbT/ pUT18C-RsbSDN2	This study
44.	BTHRG43	BTH101/pKT25-RsbT/ pUT18C-RsbSDC1	This study
45.	BTHRG44	BTH101/pKT25-RsbT/ pUT18C-RsbSDC2	This study
46.	BTHRG45	BTH101/pKT25-RsbR-mCherry/pUT18C-RsbS-GFP	This study
47.	BTHRG46	BTH101/pKT25-RsbR-mCherry/pUT18C-RsbS	This study
48.	BTHRG47	BTH101/pKT25-RsbR/ pUT18C-RsbS-GFP	This study
49.	BTHRG48	BTH101/pKT25ZIP/pUT18C-ZIP	This study

Table S5

		**Diffused (D)**	**Polar (P)**	**Bipolar (B)**	**Scattered (S)**	**% of total cells with foci (P+B+S)**	**Total cells counted (N)**
mCh		92	0	0	0	0	1295
RsbR_mCh	Exponential	93	5.3 (78%)	0.3 (4.4%)	1.2 (17.6%)	6.8	755
	Stationary	76	24 (100%)	0 (0%)	0 (0%)	24	100
GFP		100	0	0	0	0	755
RsbS_GFP	Exponential	0	40 (76%)	2.5 (4.8%)	10 (19%)	52.5	1012
	Stationary	0	51.5 (51.5%)	7 (7%)	41.5 (41.5%)	100	100

Table S6

		%Diffused (D)	%Polar (P)	%Bipolar (B)	%Scattered (S)	% total cells with foci (P+B+S)	Total cells counted
RsbR_antibody	Exponential	0	8 (31)	1 (3)	16 (66)	25	982
	Stationary	0	10 (36)	5 (13)	18 (51)	33	5956
RsbS_anti-FLAG	Exponential	0	12 (32)	1 (3)	27 (65)	40	982
	Stationary	0	13(48)	3 (14)	10 (38)	26	3246

Table S7

	%Diffused (D)	%Polar (P)	%Bipolar (B)	%Scattered (S)	% of total cells with foci (P+B+S)	Avg. total of cells counted
RsbS_DN1	52	10 (63.3)	1 (5.3)	5 (15)	16	606
RsbS_DN2	7	34 (66)	1 (2.3)	1 (23)	36	845
RsbS_DC1	36.4	15 (60)	1 (4)	8.4 (34)	24.4	556
RsbS_113N	100	0	0	0	0	635

Table S8

	%Polar	%Non-polar	%In complex	Total cells with foci
**Exponential**				
RsbS FLAG tag	31±3.2	68±3.4	21±1.0	371
RsbR Antibody	30±2.4	69±2.4	32±3.0	246
RsbR+RsbS	36±5.6	63±5.6	100±0.0	79
**Stationary**				
RsbS FLAG tag	65±4.6	34±5.0	78±0.9	1267
RsbR Antibody	64±4.4	35±4.4	81±0.1	1154
RsbR+RsbS	66±4.4	33±4.3	100±0.0	936

Table S9a Small and Big Foci

		Small foci			Big foci			Total no. of foci	Total no. of cells with foci	Avg.no of foci/cell	
		polar fraction	non-polar fraction	total %	polar fraction	non-polar fraction	total %				
RsbR	Exponential	31.7 (33.7)	62.4 (66.3)	94.1	1.4 (24)	4.5 (76.3)	6	290	179	1.6	~2
	Stationary	43.26 (51)	42 (49)	85.2	9.8 (67)	5 (33)	15	386	336	1.2	~1
RsbS_FLAG	Exponential	27 (28.5)	67.2 (71.5)	94	1.3 (22)	4.6 (78)	6	458	233	2	~2
	Stationary	44.4 (59)	31.1 (41.2)	75.5	16.5 (68)	8(32.3)	24.4	418	380	1.1	~1
RsbR+RsbS	Exponential	25.4 (27)	68.6 (73)	94.	2 (33.3)	4 (66.6)	6	51	40	1.2	~1
	Stationary	35.3 (62.3)	21.4 (37.7)	56.5	32 (74.2)	11.2 (26)	43.4	295	269	1.1	~1

Table S9b Percentage of cells with yellow foci

	Only 1 spot	Only 2 spots	>2 spots	Total % cells with yellow foci	No. of cells
Expo	5.8±1	0.8±0.21	1.3±0.24	47.7±1.5	350-600
Stat	3.4±1	0.2±0.1	0±0	6.5±2	2000-3000

Figure S1 Core gene phylogentic trees and absence/ presence of the RsbRST, RsbKMY and RsbPQ modules.

Mycobacterium species phylogeny based on 56 core genes present in all 244 indicated mycobacteria. The presence of Rsb genes in *Mycobacterium* spp. were based on *Mmar*^{T} and *M. gilvum* Rsb homologs involved in activation of the alternative sigma factor σ^{F} (Pettersson *et al*., 2013; de Been *et al*., 2011)[19,22]. Species in red represent to slow growing mycobacteria (SGM) and those marked in green correspond to rapid growing mycobacteria (RGM). Grey marked mycobacteria correspond to those for which no growth rate data could be obtained. The color boxes represent *sigF* and type of *rsb* genes and their presence as indicated (white boxes mark absence of gene). The phylogenetic tree was generated using the ITOLv3 tool[50] (see Methods).

Fig S1

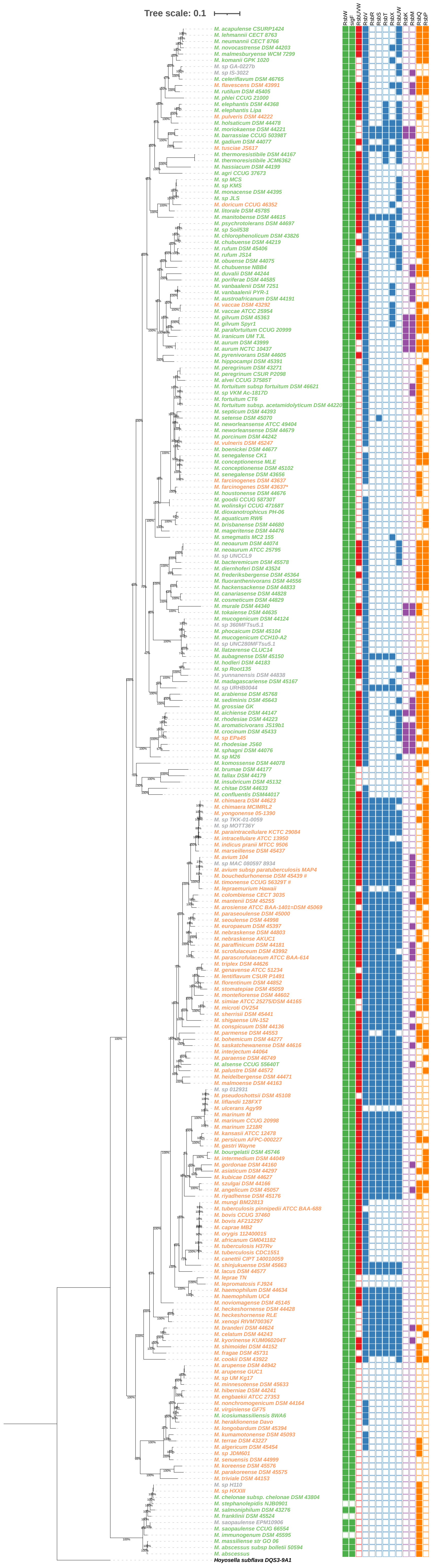

Tree scale: 0.1
RsbW
sigF
RsbUVW
RsbV
RsbR
RsbS
RsbT
RsbX
RsbUW
RsbK
RsbM
RsbQ
RsbP
Hoyosella subflava DQS3-9A1

Figure S2 Western *rsbK* and *rsbM* gene synteny in RGM.

Homologs of the *rsbK* and *rsbM* genes in sixteen RGM strains based on *Mycobacterium gilvum* PYR-GCK, *rsbK* gene (Mflv_1000) and *rsbM* gene (Mflv_1001), while Mflv_0999 encodes a response regulator receiver modulated diguanylate cyclase/phosphodiesterase with a PAS/PAC sensor(s). The vertical brown colored boxes mark homologous regions, while red arrows mark the *rsbK* and *rsbM* homologs. The *rsbK* gene encode a hybrid sensor kinase, *rsbM* a methyl-accepting chemotaxis protein and *rsbY* a PP2C serine phosphatase and these proteins constitute the RsbMKY module in *B. cereus* (Chen *et al*., 2012)[61]. The figure was generated using the genoPlotR R-package[51].

Figure S3 Structural domains in RsbK.

Domain organization of the RsbK protein in seventeen RGM strains compared to the *Bacillus cereus* RsbK protein as indicated. The figure was generated using the ITOLv3 tool[50].

Fig S2

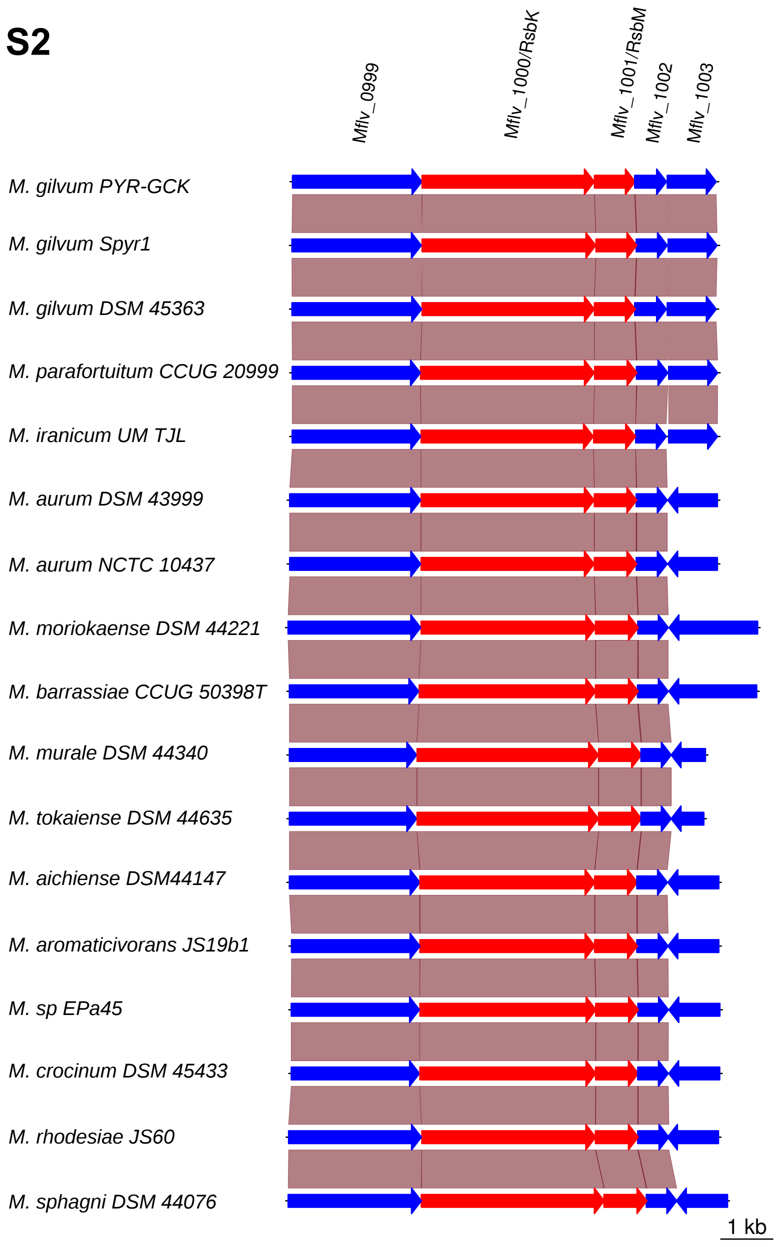
Mflv_0999
Mflv_1000/RsbK
Mflv_1001/RsbM
Mflv_1002
Mflv_1003
M. gilvum PYR-GCK
M. gilvum Spyr1
M. gilvum DSM 45363
M. parafortuitum CCUG 20999
M. iranicum UM TJL
M. aurum DSM 43999
M. aurum NCTC 10437
M. moriokaense DSM 44221
M. barrassiae CCUG 50398T
M. murale DSM 44340
M. tokaiense DSM 44635
M. aichiense DSM44147
M. aromaticivorans JS19b1
M. sp EPa45
M. crocinum DSM 45433
M. rhodesiae JS60
M. sphagni DSM 44076
1 kb

Fig S3

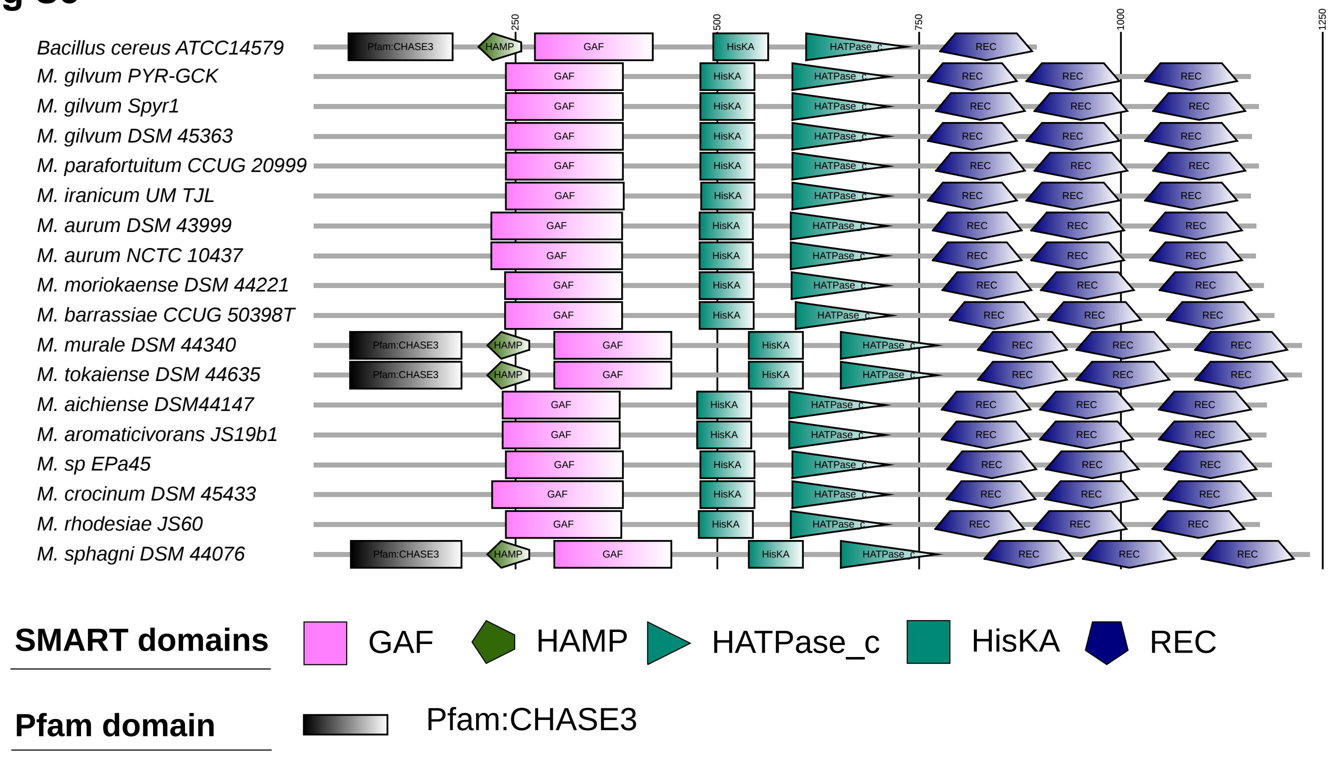
250
500
750
1000
1250
Bacillus cereus ATCC14579
M. gilvum PYR-GCK
M. gilvum Spyr1
M. gilvum DSM 45363
M. parafortuitum CCUG 20999
M. iranicum UM TJL
M. aurum DSM 43999
M. aurum NCTC 10437
M. moriokaense DSM 44221
M. barrassiae CCUG 50398T
M. murale DSM 44340
M. tokaiense DSM 44635
M. aichiense DSM44147
M. aromaticivorans JS19b1
M. sp EPa45
M. crocinum DSM 45433
M. rhodesiae JS60
M. sphagni DSM 44076
Pfam:CHASE3
HAMP
GAF
HisKA
HATPase_c
REC
SMART domains
GAF
HAMP
HATPase_c
HisKA
REC
Pfam domain
Pfam:CHASE3

Figure S4 "STAS" domains and protein-protein interactions in the RsbRST-complex. (a – d) The *E. coli* strain BTH101 was transformed with various pairwise constructs as indicated and as described above. Blue color was scored as a positive interaction. RsbR, RsbS and RsbT interacted with each other as also shown previously (Pettersson *et al*., 2013)[22]. RsbR also interacts with itself and large deletions appeared not to affect the interaction. Note that deletion of the RsbR (DN5) or RsbS STAS domains resulted in a loss of RsbR-RsbS interaction but see below and the main text. Also, the BATCH data did not suggest that RsbT interacts with any of the deletion variants of RsbR or RsbS. Table 1 in the main text summarize the data.

(e) Schematic representation of the co-immunoprecipitation constructs as indicated. R, S and T refer to RsbR, RsbS and RsbT, respectively, while RDC1, RDN4, RDN5, and SDN1 represent different RsbR and RsbS deletion constructs. The different domains are marked as shown in the figure.

(f) Schematic representation of the RsbR, RsbS and RsbT co-expression constructs (corresponding to Table S2d). The domains are marked as in (e).

(h) Western blot analysis of co-immunoprecipitated proteins of the one replicate (see also Table 1). The anti-FLAG antibody was used to pull down protein complexes from lysates of RsbR-, RsbS-, and RsbT-expressing (including deletions) *E. coli* BL21 DE3 cells. After purification of complexes the eluted proteins were analysed by Western blotting using anti-FLAG antibodies (top panels) and anti-6xHis antibodies (bottom panels; see Supplementary methods for details). M indicates that a PageRuler plus pre-stained marker (to the left and right of the blots) was used to estimate protein sizes (in kDa). The top and bottom blots originate from same filter, the difference being exposure to different excitation wavelengths (see Supplementary methods for details). R, S and T refer to RsbR, RsbS and RsbT, respectively, while RDC1, RDN4, RDN5, and SDN1 represent different RsbR and RsbS

deletion constructs (see text for details). The order of the FLAG/His represents whether the tags are N-terminal or C-terminal fusions, *e.g*. S-FLAG-His-R (lane 4) indicates that the FLAG-tag is fused to the C-terminus of RsbS and the 6xHis tag is fused to the N-terminus of RsbR. Table 1 in the main text summarize the data.

Fig S4

a RsbR(pUT18)+RsbR deletions (pKT25)

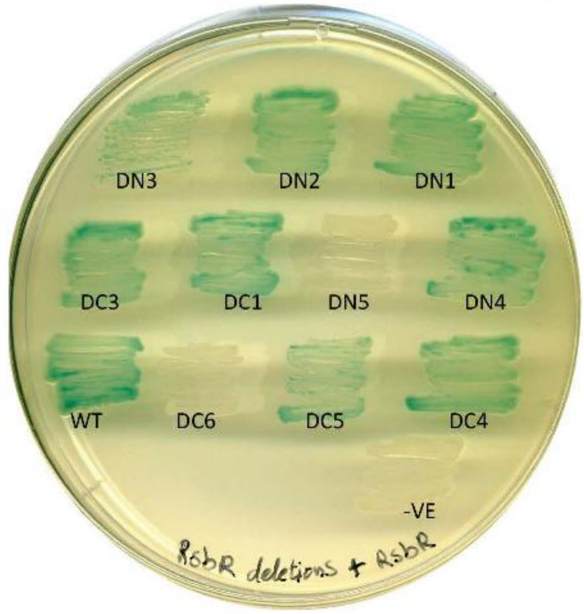

b RsbS(pUTI8C)+RsbR deletions (pKT25)

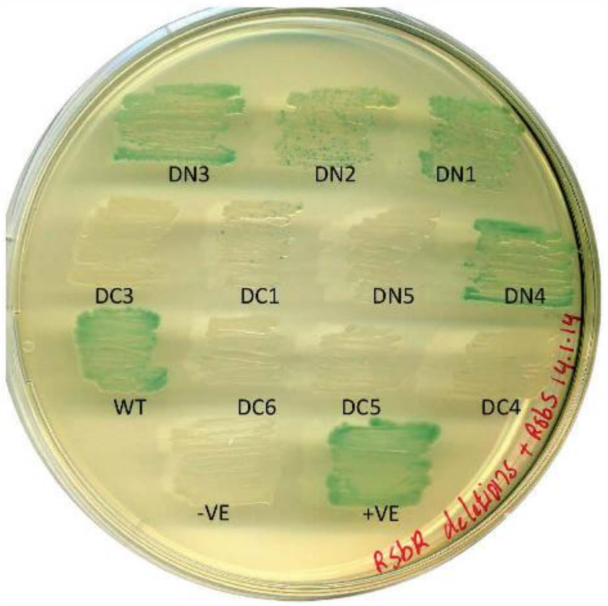

c RsbT(pUT18)+RsbR deletions (pKT25)

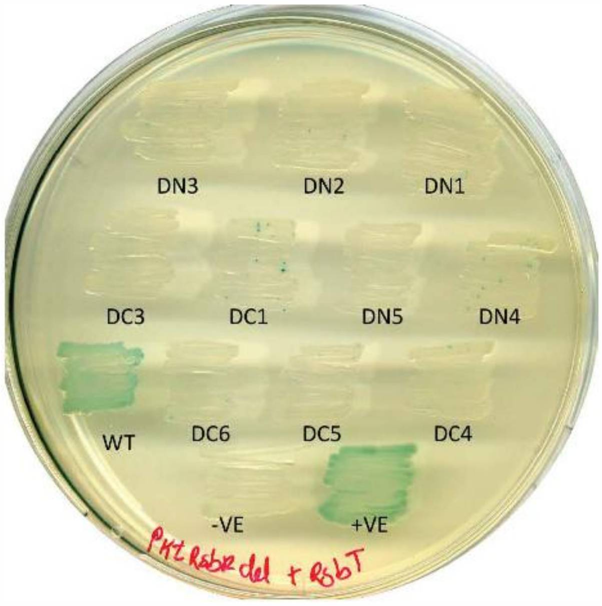

d

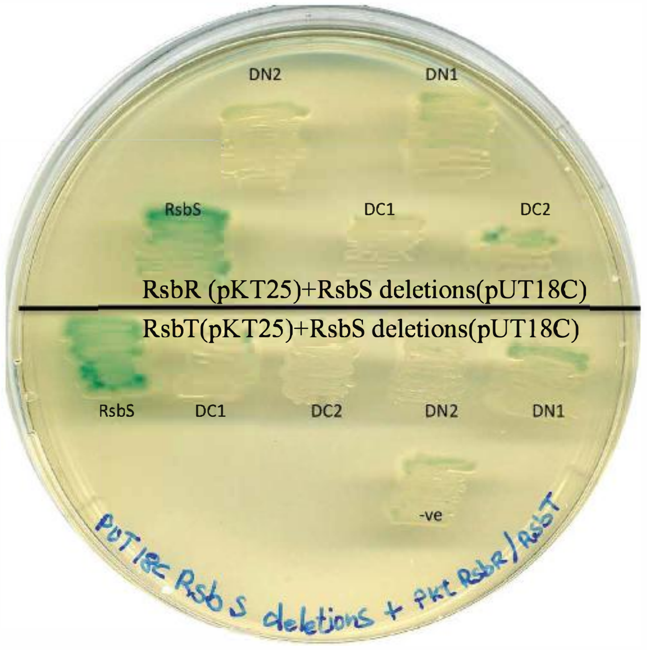

e

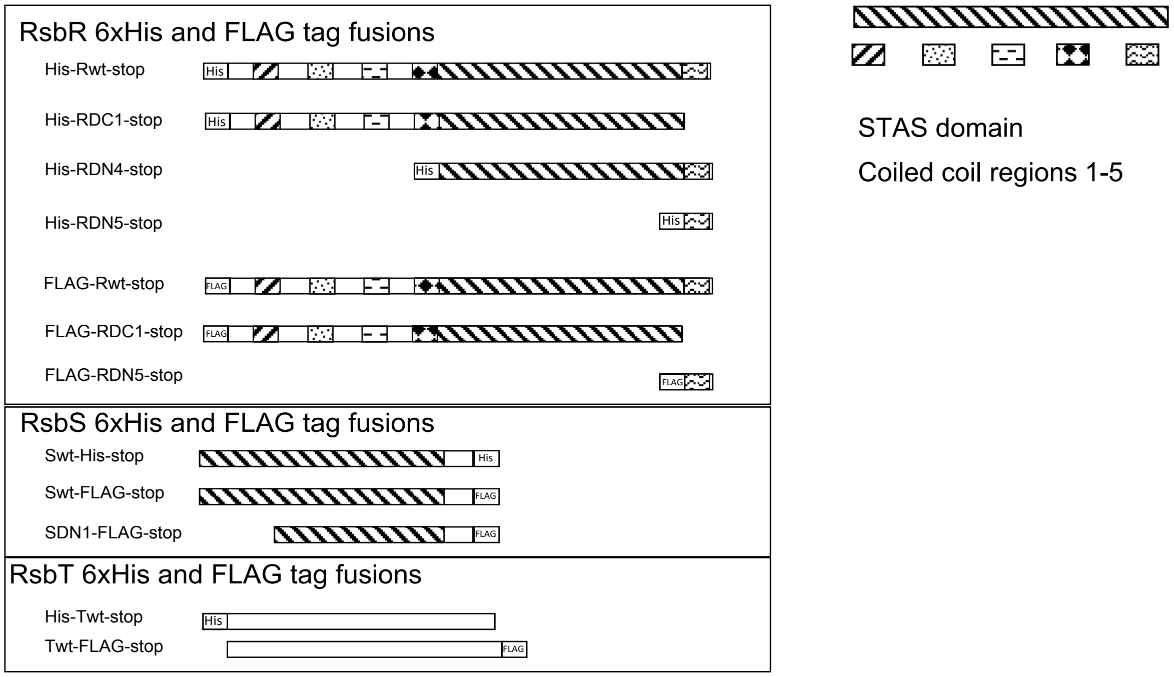
RsbR 6xHis and FLAG tag fusions
His-Rwt-stop
His-RDC1-stop
His-RDN4-stop
His-RDN5-stop
FLAG-Rwt-stop
FLAG-RDC1-stop
FLAG-RDN5-stop
RsbS 6xHis and FLAG tag fusions
Swt-His-stop
Swt-FLAG-stop
SDN1-FLAG-stop
RsbT 6xHis and FLAG tag fusions
His-Twt-stop
Twt-FLAG-stop
STAS domain
Coiled coil regions 1-5

f

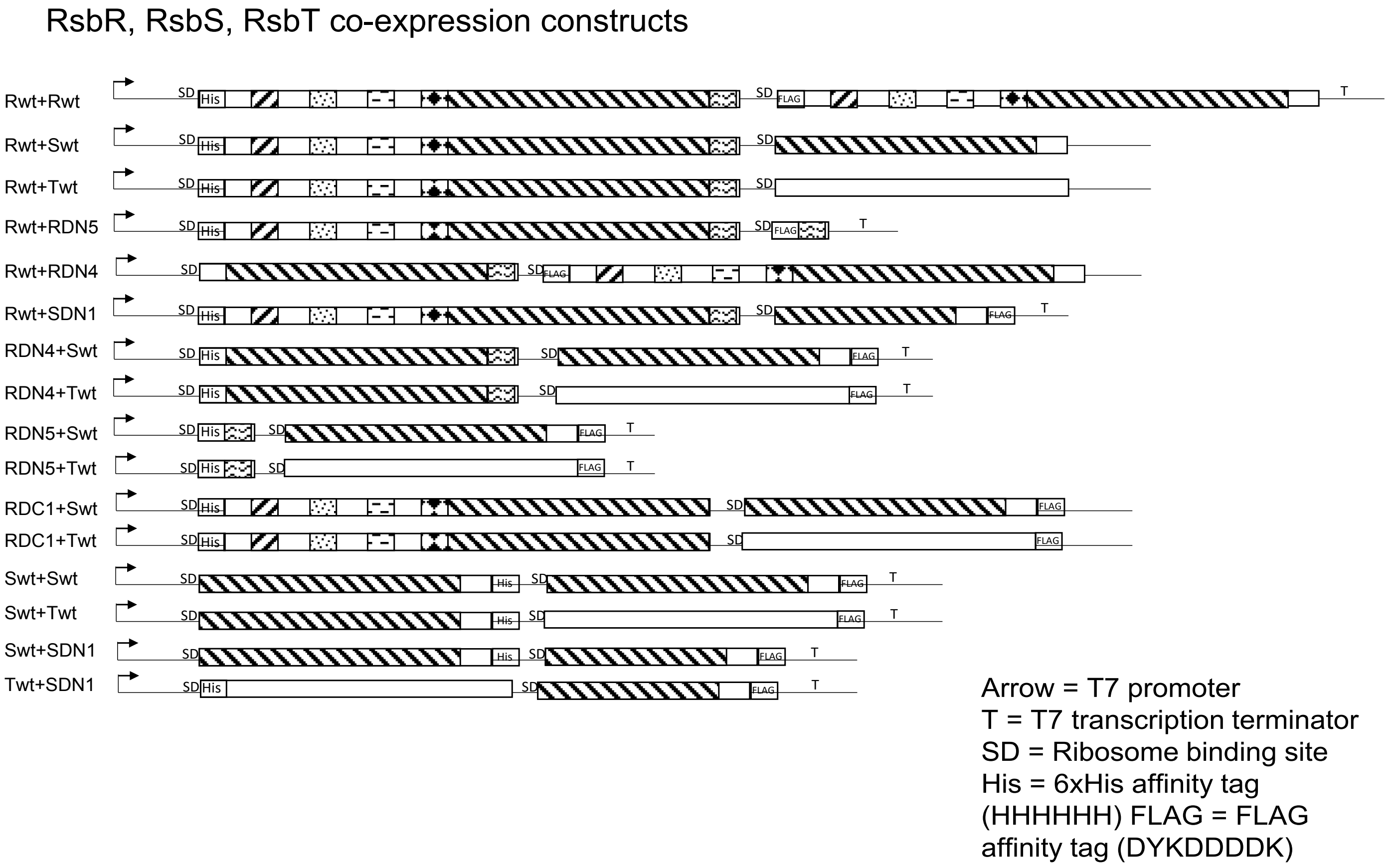
RsbR, RsbS, RsbT co-expression constructs
Rwt+Rwt
Rwt+Swt
Rwt+Twt
Rwt+RDN5
Rwt+RDN4
Rwt+SDN1
RDN4+Swt
RDN4+Twt
RDN5+Swt
RDN5+Twt
RDC1+Swt
RDC1+Twt
Swt+Swt
Swt+Twt
Swt+SDN1
Twt+SDN1
Arrow = T7 promoter
T = T7 transcription terminator
SD = Ribosome binding site
His = 6xHis affinity tag (HHHHHH) FLAG = FLAG affinity tag (DYKDDDDK)

g

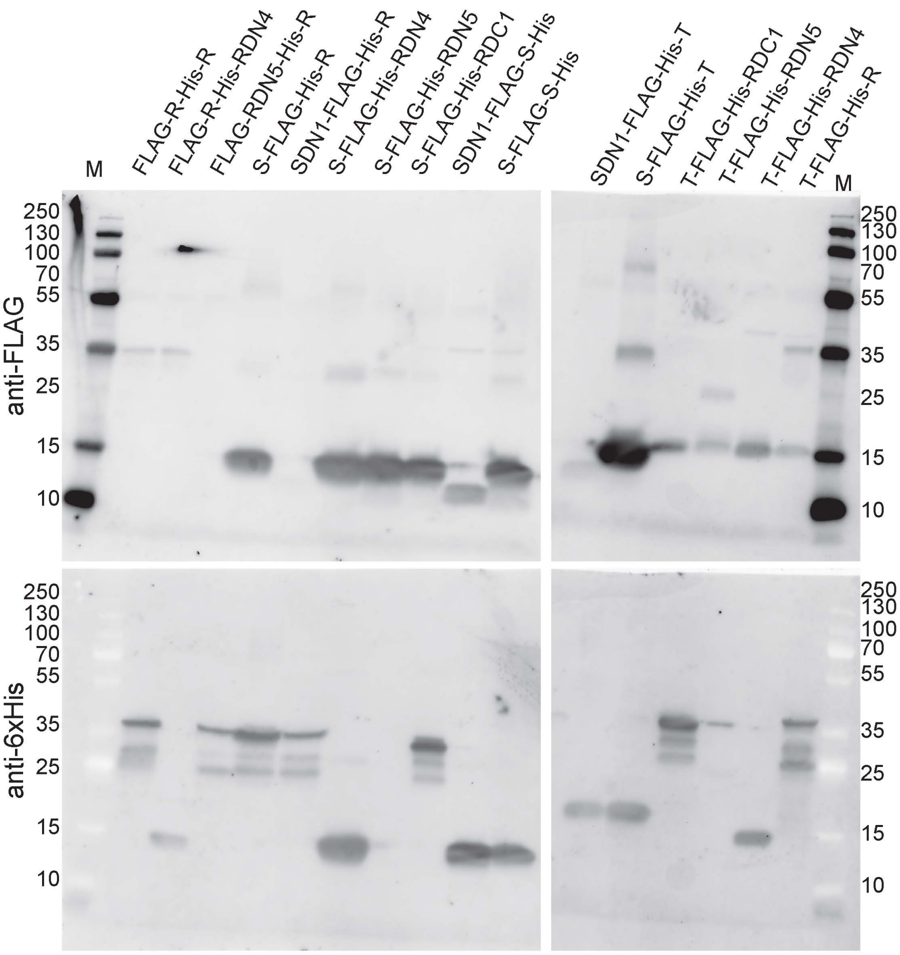
M
FLAG-R-His-R
FLAG-R-His-RDN4
FLAG-RDN5-His-R
S-FLAG-His-R
SDN1-FLAG-His-R
S-FLAG-His-RDN4
S-FLAG-His-RDN5
S-FLAG-His-RDC1
SDN1-FLAG-S-His
S-FLAG-S-His
SDN1-FLAG-His-T
S-FLAG-His-T
T-FLAG-His-RDC1
T-FLAG-His-RDN5
T-FLAG-His-RDN4
T-FLAG-His-R
M
anti-FLAG
anti-6xHis
250
130
100
70
55
35
25
15
10

Figure S5 Western blot analysis showing the expression of RsbR-mCherry and RsbS-GFP in *Mmar*^{T}.

(a) Western blot using mCherry primary antibodies and horseradish peroxidase (HRP) tagged secondary antibodies. The chemiluminescence signal was detected by a CCD camera attached to a chemiDoc system (BioRad). The lysates from *Mmar*^{T} cells transformed with different constructs were loaded: lane 1 pST2K-RsbR-mCherry-RsbS-GFP; lane 2 pBS401-RsbR-mCherry; lane 5 pBS401-mCherry. Lane 3 and lane 4 show lysates from two unrelated deletions of RsbR-mCherry, which also did not show polar localization (data not shown). Note that the size of the RsbR-mCherry fusion protein is bigger in lane 1 compared to lane 2 due to presence of the His-6 tag at the 'N' terminus. The expected size of RsbR-mCherry fusion protein is ≈ 60 kDa. The mCherry alone has a size of approximately 26 kDa. The fusion protein and the mCherry protein are marked with white arrows.

(b) Western blot using GFP antibodies. Lanes 1 and 2 were loaded with the same lysates as in lanes 1 and 2 in panel A. The expected size for RsbS-GFP fusion is ≈43 kDa. The fusion protein and the GFP protein are marked with white arrows.

The molecular standards (Thermo Scientific Page Ruler Plus Ladder; Thermo Fisher) were loaded in lane M in both as indicated in both panel A and B. The percentages of fusion products (upper band) are shown below each panel. The protein quantitation was performed using ImageJ software.

Fig S5

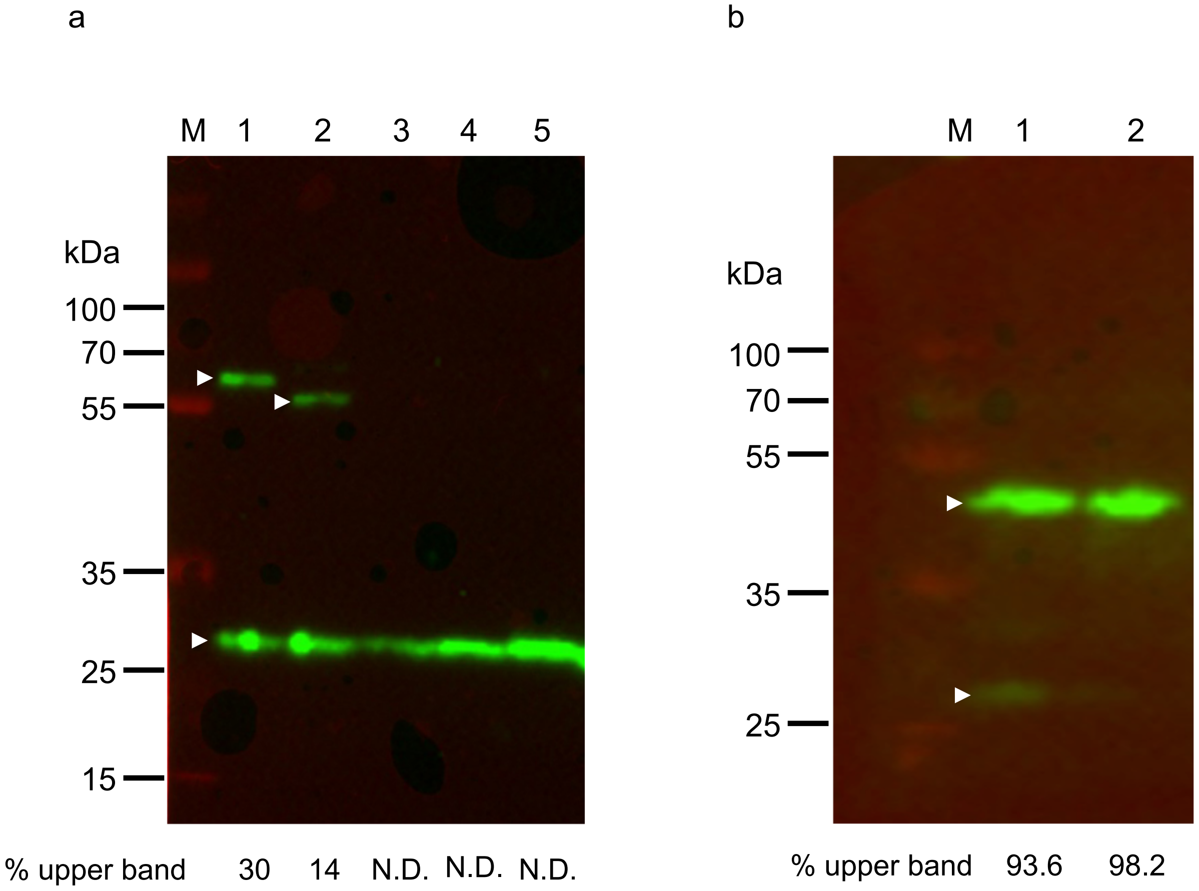

a
M 1 2 3 4 5
kDa
100
70
55
35
25
15
% upper band 30 14 N.D. N.D. N.D.
b
M 1 2
kDa
100
70
55
35
25
% upper band 93.6 98.2

Figure S6 Sample line profiles obtained quantifying the fluorescence along *Mmar*^{T} cells expressing both RsbR-mCherry and RsbS-GFP.

(a) Representative line profiles obtained by plotting the fluorescence intensity (arbitrary unit) along the *Mmar*^{T} cell length (expressed in % cell length) for cells expressing RsbR-mCherry and RsbS-GFP. Clear peaks were observed at one of the cell poles for cells expressing RsbR-mCherry or RsbS-GFP compared to cells expressing mCherry or GFP.

(b) Shows the pre-immune sera treated *Mmar*^{T} cells, which served as a control. The second panel shows the background signal when antibodies against RsbS was used.

Fig S6

a

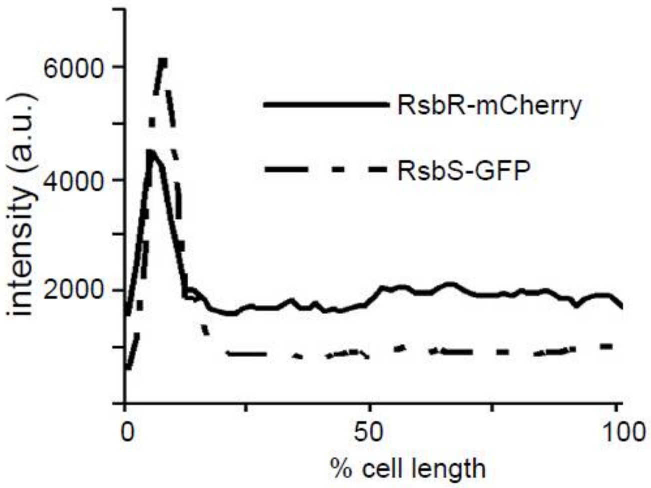

b

RsbR antibody-FITC RsbS antibody-Alexa red

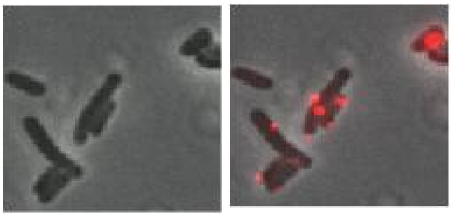

Figure S7 Bacterial two hybrid (BACTH) assay with RsbR-mCherry and RsbS-GFP constructs.

The pKT25-RsbR-mCherry and pUT18c-RsbS-GFP were transformed pair wise as indicated and described in supplementary methods. Blue colonies indicate positive interaction.

(a) BACTH interactions as indicated: 1. RsbR-mCherry+RsbS-GFP, 2. RsbR-mCherry+RsbS, 3. RsbR+RsbS-GFP, 4. RsbR+RsbS, 5. pKT25+pUT18c, 6. RsbR-mCherry+RsbS-GFP, 7. RsbR-mCherry+RsbS-GFP. Three different clones of RsbR-mCherry were tested marked 1, 6 and 7 and all three were scored as positive.

(b - d) Confirmation of the presence of the different proteins as indicated by western blotting. Lanes 1 and 2; total cell extract from colony 4 and colony 1, respectively. See above Figure S6 Panel (a). M = the SDS-PAGE ladder. Note that RsbR-mCherry (lane 2 in b and c) migrated slower due to its larger size (~95 kDa) compared to RsbR without mCherry (lane 1 in b). RsbS-GFP fusion was detected with a GFP antibody (d).

Fig S7

a

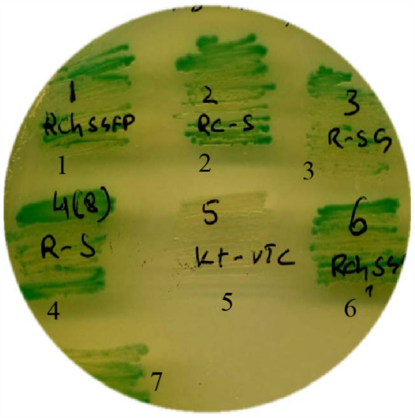

1. pKT25RsbR-mCherry + pUT18cRsbS-GFP
2. pKT25RsbR-mCherry + pUT18cRsbS
3. pKT25RsbR + pUT18cRsbS-GFP
4. pKT25RsbR + pUT18cRsbS
5. pKT25 + pUT18c
6. pKT25RsbR-mCherry + pUT18cRsbS-GFP
7. pKT25RsbR-mCherry + pUT18cRsbS-GFP

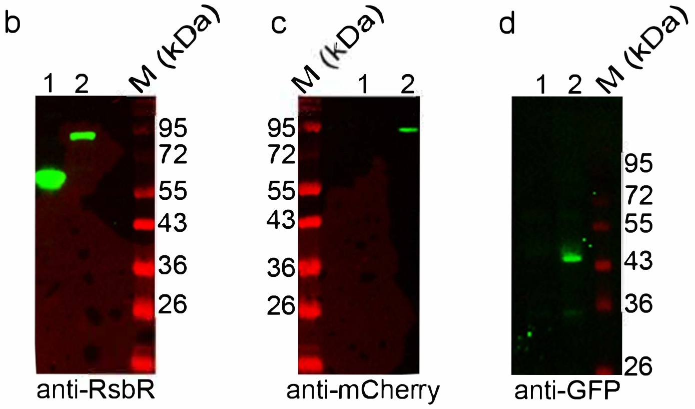

Figure S8 Fluorescence microscopy data corresponding to each main figure showing all channels recorded.

Images of different channels corresponding to each panel shown in the main figures as indicated.

Images referred to Fig 2a: Red (mCherry fluorescence), Phase (no colour) and green (GFP fluorescence).

Images referred to Fig 3a: Red (RsbS-Anti FLAG immunofluorescence), Phase (no colour), and green (RsbR antibody immunofluorescence).

Images referred to Fig 4a: Green (GFP fluorescence) and Phase (no colour).

Images referred to Fig 5c:

RsbS-GFP+RsbR-mCh (Exponential and Stationary): Green (GFP fluorescence), Red (mCherry fluorescence), overlay (red, green and yellow) and Phase (no colour).

RsbR-Antibody + RsbS-FLAG tag antibody (Exponential and Stationary): Green (RsbR-antibody Immunofluorescence), Red (RsbS-Anti-FLAG immunofluorescence), overlay (red, green and yellow antibody Immunofluorescence) and Phase (no colour).

Fig S8

Images refer to Fig 2a

RsbR-mCh_Exponential

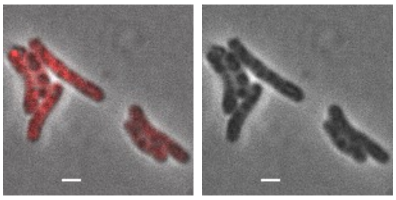

RsbR –mCh_Stationary

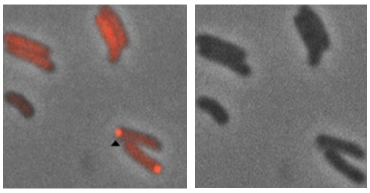

RsbS-GFP_Exponential

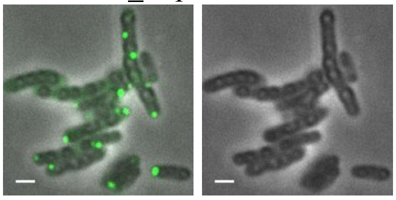

RsbS-GFP_Stationary

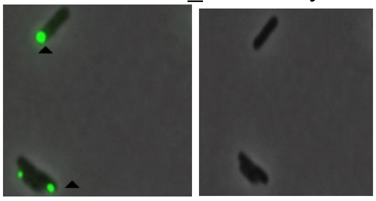

Images refer to Fig 3a

RsbS-FLAG_Exponential

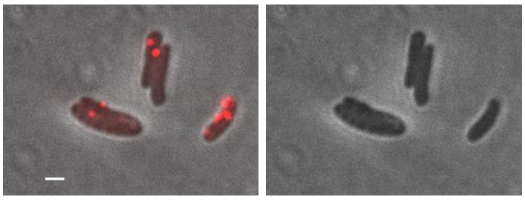

RsbS-FLAG_Stationary

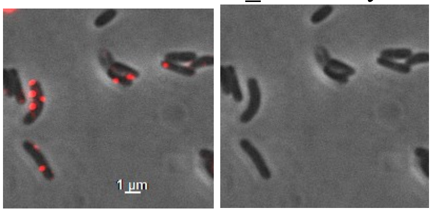

RsbR-Exponential

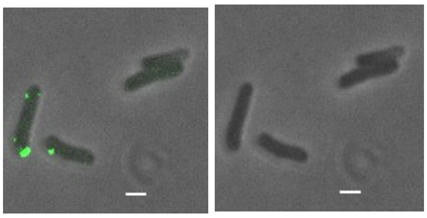

RsbR_Stationary

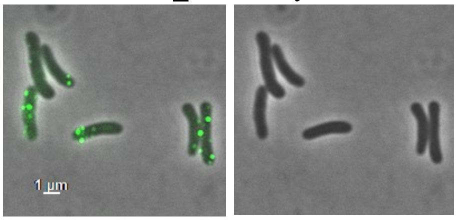

Images refer to Fig 4a

RsbS-DN1

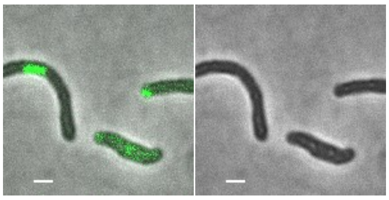

RsbS-DN2

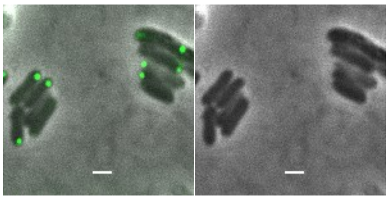

RsbS-113N

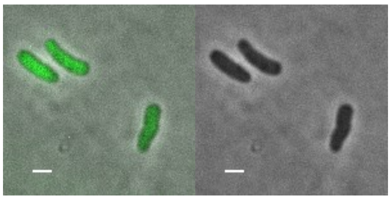

RsbS-DC1

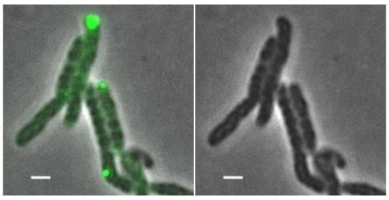

Images refer to Fig 5c

RsbS-GFP+RsbR-mCh_Exponential

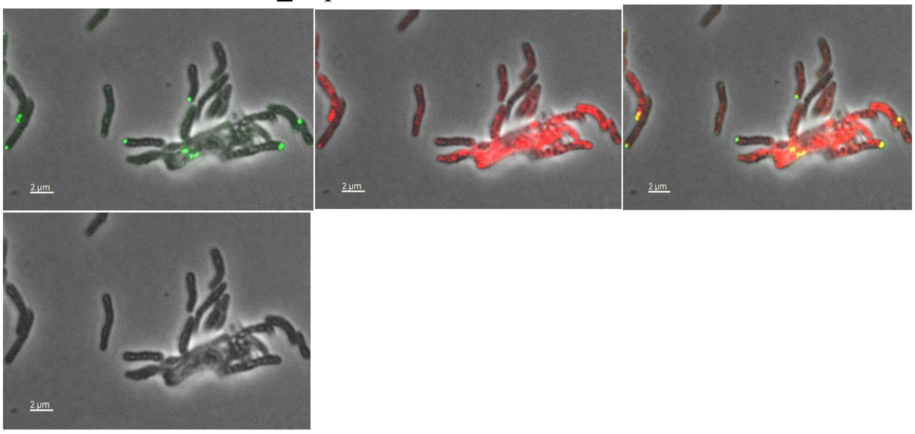

RsbS-GFP+RsbR-mCh_Stationary

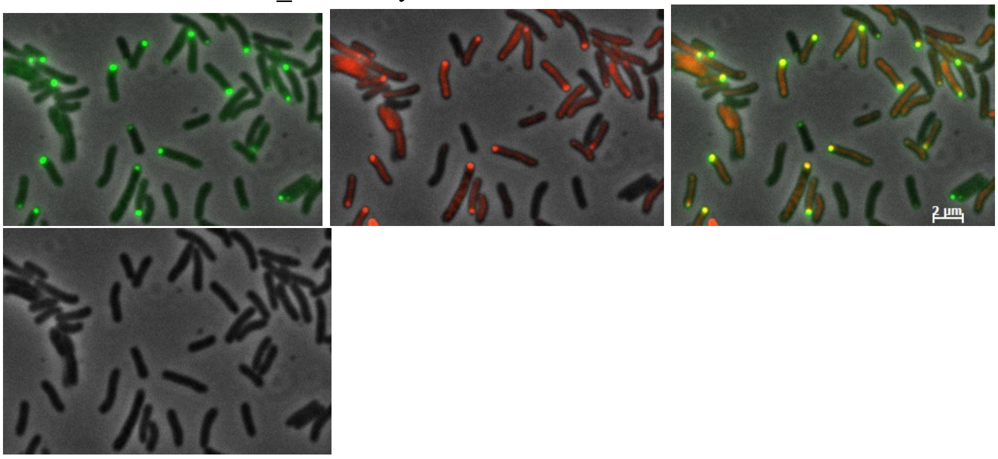

RsbR-Antibody + RsbS-FLAG tag antibody_ Exponential

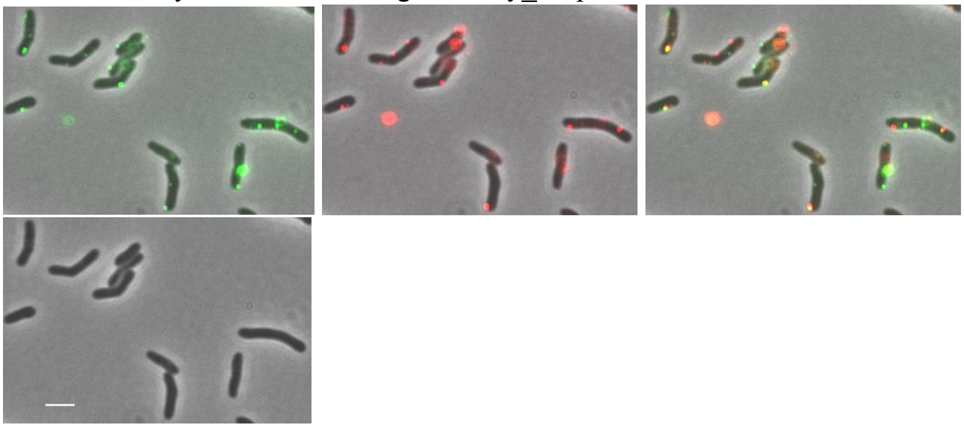

RsbR-Antibody + RsbS-FLAG tag antibody_ Stationary

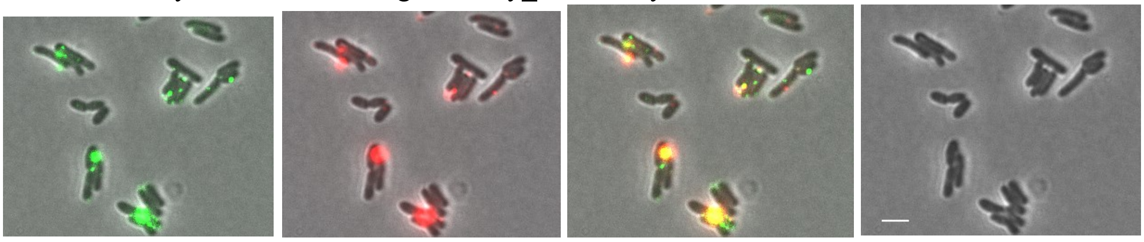

Supplement: Supplementary file 1 — Supplementary Information [file 41598_2021_89069_MOESM1_ESM.pdf]
